# Gene loss during a transition to multicellularity

**DOI:** 10.1038/s41598-023-29742-2

**Published:** 2023-03-31

**Authors:** Berenice Jiménez-Marín, Jessica B. Rakijas, Antariksh Tyagi, Aakash Pandey, Erik R. Hanschen, Jaden Anderson, Matthew G. Heffel, Thomas G. Platt, Bradley J. S. C. Olson

**Affiliations:** 1 Division of Biology, Kansas State University, Manhattan, KS 66506 USA; 2 Interdepartmental Genetics Graduate Program, Kansas State University, Manhattan, KS 66506 USA; 3 Los Alamos National Lab, Los Alamos, NM 87545 USA

**Keywords:** Genome informatics, Genomics

## Abstract

Multicellular evolution is a major transition associated with momentous diversification of multiple lineages and increased developmental complexity. The volvocine algae comprise a valuable system for the study of this transition, as they span from unicellular to undifferentiated and differentiated multicellular morphologies despite their genomes being similar, suggesting multicellular evolution requires few genetic changes to undergo dramatic shifts in developmental complexity. Here, the evolutionary dynamics of six volvocine genomes were examined, where a gradual loss of genes was observed in parallel to the co-option of a few key genes. Protein complexes in the six species exhibited novel interactions, suggesting that gene loss could play a role in evolutionary novelty. This finding was supported by gene network modeling, where gene loss outpaces gene gain in generating novel stable network states. These results suggest gene loss, in addition to gene gain and co-option, may be important for the evolution developmental complexity.

## Introduction

The evolution of multicellular organisms is a prerequisite for the emergence of complex organismal body plans^1,2^. However, the genomic basis for multicellular evolution is poorly understood^3^. The volvocine algae are a valuable model system for studying multicellular evolution because they have undergone a recent transition to multicellularity (~ 200 MYA)^4^, and the ~ 100 member species span a wide range of developmental complexity within a size range from 10 µm to 3 mm^5^. This group includes unicellular *Chlamydomonas*, undifferentiated multicellular bowl-shaped *Gonium* and spheroidal *Pandorina* (8–16 cells), multicellular isogamous *Yamagishiella* (32 cells)*,* multicellular anisogamous *Eudorina* (32 cells), and multicellular differentiated *Volvox* (> 500 cells)^6,7^.

**Figure 1:**
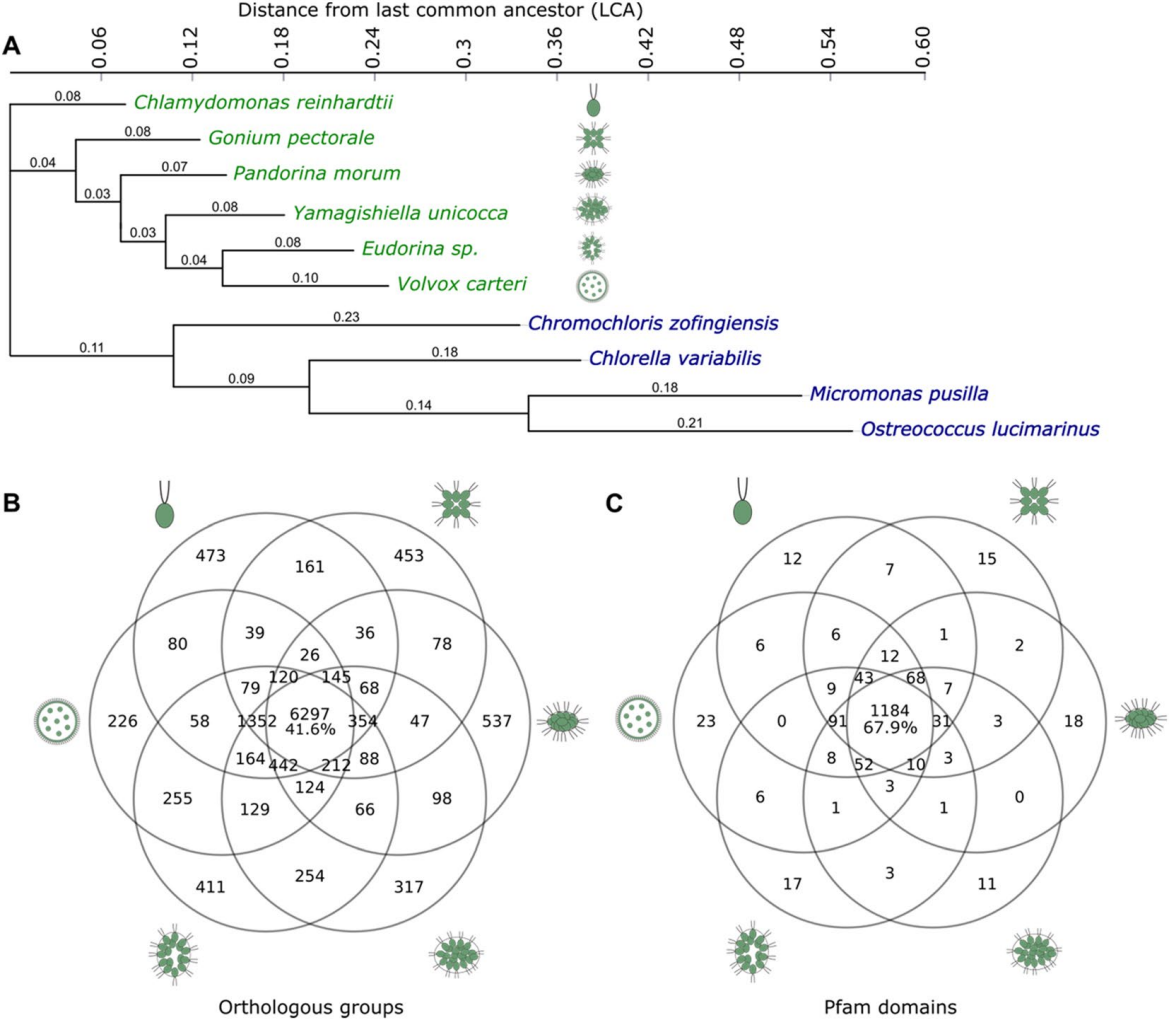
Volvocine algal genomes are similar. (**A**) Genome-wide phylogeny of the volvocine algae (green) compared to other chlorophyte outgroups (blue) inferred by PosiGene. Branch values indicate distance from the nearest node. Note *Chlamydomonas* is unicellular and closely related to colonial volvocines. (**B**) Orthologous groups shared between the six volvocine species (Supplementary File 1). (**C**), Pfam domains shared between six volvocine species.

Based on morphology, differentiated multicellular volvocines were predicted to have evolved from their unicellular ancestors by stepwise acquisition of developmental processes, such as establishment of organismic polarity, genetic control of cell number, size expansion, and division of labor among cell lineages^6^ (Fig. 1A, cartoons). However, phylogenetic approaches suggest that diversification among the volvocine algae was rapid^4^ and many of the genetic changes allowing for the evolution of developmental complexity took place early in the evolution of the volvocines. This suggests that genetic gains for each step might not fully explain the organismal complexity of the volvocine algae^8^.

It has been proposed that shared genetic toolkits and pathways that act as developmental patterning modules can lead to divergent phenotypic outputs in other multicellular lineages^9,10^. Indeed, the genomes of *Chlamydomonas reinhardtii*, *Gonium pectorale*, and *Volvox carteri* (hereafter referred to by genus) are highly similar and syntenic^8,11,12^. Moreover, a homolog of the cell cycle regulator and tumor suppressor *Retinoblastoma* (RB) is sufficient for undifferentiated multicellularity^8^, suggesting co-option of a developmental regulator can result in complex developmental patterns required for multicellularity^3^. Thus, the evolution and diversification of the volvocine algae is thought to have heavily relied on the co-option of key functional elements.

Co-option, however, is unlikely to be the only evolutionary process behind volvocine multicellularity. Gene loss is found in many lineages undergoing evolutionary change^13–17^, and it is likely a major force in eukaryotic evolution^18^ with the potential to influence evolutionary trajectories^19^, to the extent that gene loss has been proposed to be a dominant force in genome evolution overall^20^. Nevertheless, its action in concert with other evolutionary drivers is not well understood^21–24^. The study of gene loss is challenging because loss events can be the result of trait evolution (e.g. through relaxed selection), but they can also cause phenotypic adaptation^19^. Moreover, gene losses can impact an organism’s evolvability and speed of adaptation^25^, depending on their dispensability and position within the regulatory and protein–protein interaction network^18,25^.

To investigate whether gene loss played a role in volvocine evolution, we report the assembly of *Pandorina morum* and annotation of genomes from *P. morum, Yamagishiella unicocca* and *Eudorina sp.* (henceforth referred to by genus). These newly analyzed genomes, in addition to those of *Chlamydomonas, Gonium,* and *Volvox*^8,11,26^, were subjected to comprehensive analysis of genic evolutionary trajectory. We demonstrate that significant loss of conserved genes has occurred in the volvocine lineage, and through a gene network model, we show that gene loss outperforms gene gain in generating stable novelty. Our results additionally suggest that only a few genes were co-opted for volvocine multicellularity. Furthermore, differential protein–protein interactions (PPI) are observed between species. These results suggest that gene loss at the transition to multicellularity may impact molecular network structure such that novel functions associated to changes in developmental complexity can evolve.

## Results

### Volvocine genomes share genomic and functional toolkits

We validated the annotation of seven closely related volvocine algae exhibiting major changes in morphology (Fig. 1A cartoons) using BUSCO^6,27^. BUSCO inferences suggest that the *Chlamydomonas, Gonium, Pandorina, Yamagishiella, Eudorina,* and *Volvox* annotated genomes are of high quality (Supplementary Table S1). However, BUSCO analysis of the *Tetrabaena* genome showed it had low completeness values (63.6%). Hence, it was not used for downstream analyses despite being an available volvocine genome. Genome comparisons (Supplementary Table S2), orthologous group and protein family domain (Pfam) catalogs for the analyzed species and their chlorophyte outgroups were compiled, and a genome-wide reconstruction of volvocine phylogeny was generated (Fig. 1A) based on orthologous groups, which is in close agreement with previously determined phylogenies based on smaller gene sets^8,28,29^.

Overall, 15,155 orthologous groups were identified; of these, 6297 (41.6%) are shared among the six genomes, and 2417 are species specific (15.9%, Fig. 1B, Supplementary Table S3). Analysis of identified Pfams^30^ in the six species shows 1743 Pfam domains. Of these, 1184 (67.9%) are shared in all six species, and 96 (5.5%) are species specific. Notably, 8922 (58.9%) orthologous groups, and 1479 (84.9%) Pfams are shared by at least 5 species (Fig. 1C). These results support previous findings of minimal species-specific genomic and functional toolkits between the volvocine genomes^8,26^, despite their marked developmental and morphological differences.

### Contraction outpaces expansion of conserved genetic and functional elements as complexity increases

**Figure 2:**
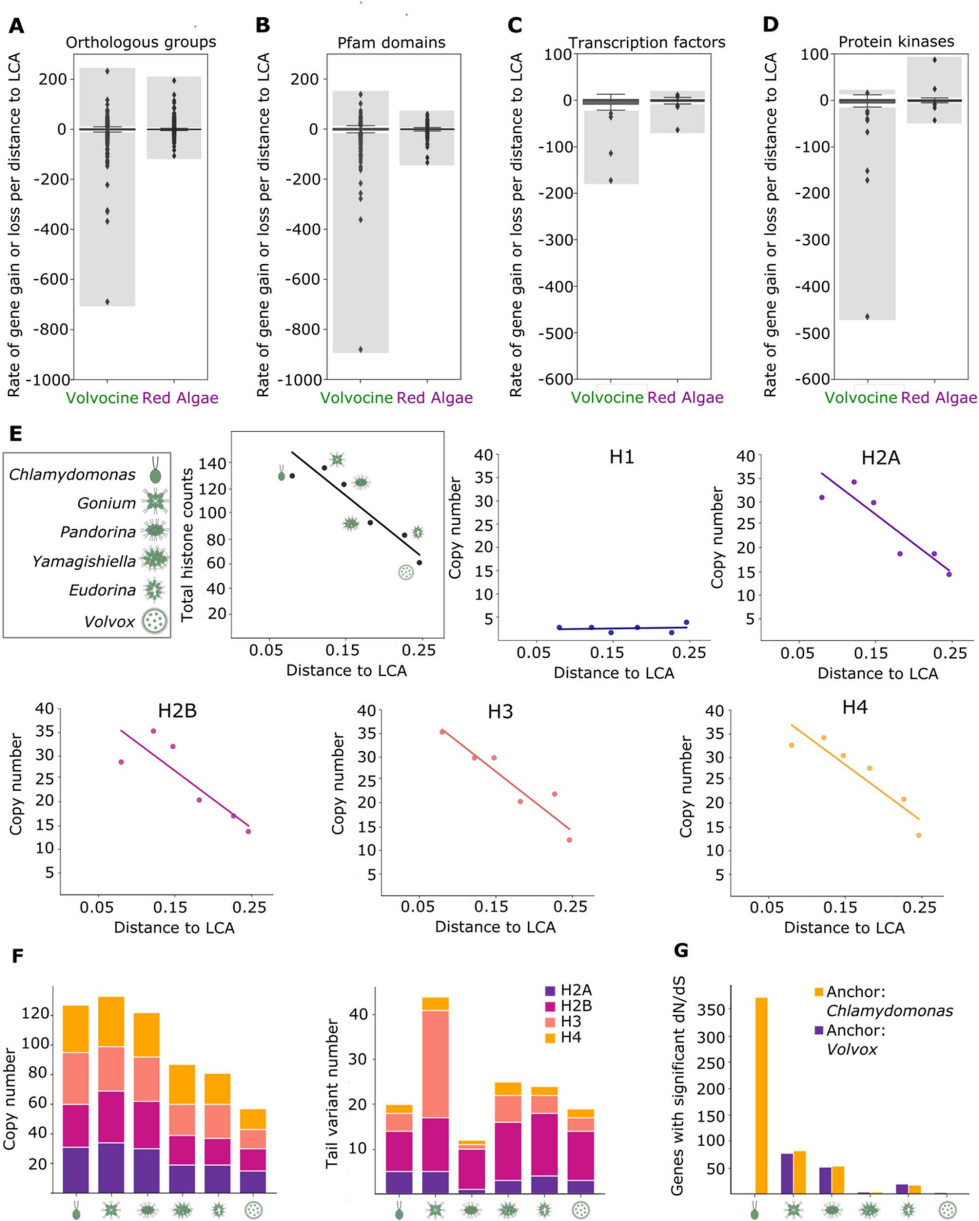
Gene loss outpaces gain in volvocine algae genomes. Distribution of the gene loss and gain rates per distance to LCA in the volvocine genomes compared to Rhodophyceae (red algae) of (**A**) orthologous groups, (**B)** Pfam domains, (**C**) transcription factors, and (**D**) protein kinases. Outlier groups, which represent significant loss (negative values) or gain (positive values) rates, are within the shaded grey area. (**E**) Histone copy number relative to distance to LCA shows a decreasing trend except for H1 in the volvocine genomes. Top left panel describes total histone counts per species. (**F**) Histone copy numbers (left) are reduced with minimal loss of tail variants (right). (**G**) Quantification of positively selected genes per species using *Chlamydomonas* (yellow) and *Volvox* (purple) as reference genomes.

Given the similarity between volvocine algae genomes (Fig. 1B,C), we hypothesized that small differences between the *Chlamydomonas, Gonium, Pandorina, Yamagishiella, Eudorina* and *Volvox* genomes contribute to the evolution of developmental complexity. Rates of evolution of shared genes and protein domains (7942 orthologous groups and 1338 Pfam domains) for the six species were analyzed by normalization to the distance from their Last Common Ancestor (LCA) to account for potential signatures of gene or domain gain and loss. This analysis revealed that 402 (5.1%) orthologous groups have undergone significant changes, with a bias towards gene loss (176 expanding orthologous groups, 226 contracting, Fig. 2A outliers, shaded grey area). An independent approach to evaluate contraction of orthologous groups using GLOOME^31^ confirmed that 141 of the 226 contracting orthologous groups (62.4%) underwent losses at different points of volvocine evolution (Supplementary File 2). Of the remaining groups, 51 of the 226 (22.6%) cannot be subjected to GLOOME analysis (see “Methods”), which means that only 34 orthologous groups (15%) show discrepancies between both methods.

Surprisingly, while most Pfam domains were conserved, significantly expanding domains were less abundant than their contracting counterparts (51 and 127, respectively, Fig. 2B outliers, shaded grey area). Pfam domain counts per gene did not show significant variation between the six species (Supplementary Fig. S1), and contraction trends were not observed in highly conserved genes actin, β-tubulin, α-tubulin, chloroplast ferredoxin, mtATP-synthase α, mtATP-synthase β, and dynein heavy chain (Supplementary Fig. S2, Supplementary Table S4).

Co-option of master regulators such as transcription factors (TFs) or protein kinases (PKs) is thought to be important for evolutionary innovation^8,32^. Analysis of all identified TF and PK families in the volvocine algae (47 and 69 respectively) revealed that 5 families of TFs (10.6%) underwent significant contraction, while none expanded significantly (Fig. 2C, Supplementary Fig. S3A). Similarly, 10 families of PKs (14.5%) underwent significant change, where 1 of the PK families expanded and 9 contracted (Fig. 2D, Supplementary Fig. S3B). These data suggest that volvocine algae have lost genes with conserved functions, while gene gain occurred at a much lower rate and frequency (Fig. 2A–F, Supplementary Fig. S3).

To gain further insight into gene loss as a mechanism of multicellular evolution, we analyzed histone genes for their evolutionary dynamics. Chromatin is an important regulator of gene expression, where post-translational modifications of histone N-termini are conserved and well understood^33^. Thus, histone genes were analyzed for their evolutionary dynamics. All families of histones, except linker H1, were reduced in number as developmental complexity increases in the volvocine algae (Fig. 2E, Supplementary Tables S5–S6). Analysis of histone N-termini methylation site variation for H2A, H2B, H3, and H4 shows that H2A, H2B and H4 underwent copy number reduction (Fig. 2F, Supplementary Table S6). However, Histone H3 exhibited greater N-terminal variant diversity in *Gonium* (Fig. 2F, Supplementary Table S6). These data suggest that post-translational changes in histones may facilitate increases in developmental complexity through altering gene expression in the context of gene loss.

Red algae (Rhodophyceae) also display various degrees of developmental complexity and exhibit signatures of gene loss^21^. Hence, orthologous groups (Fig. 2A), Pfam domains (Fig. 2B), TFs (Fig. 2C), PKs (Fig. 2D) and histone (Supplementary Table S7) dynamics were examined in parallel to their volvocine counterparts. This analysis confirmed the presence of contraction signatures in red algae, agreeing with earlier reports^21^, as well as the presence of expansion signatures (Fig. 2D). These results validate the usefulness of a regression-based approach to identify candidate genetic and functional units with a history of expansion or contraction.

To assess the impact of positive selection on increases to developmental complexity, orthologous groups of genes with representatives in all six species were subjected to *d*N/*d*S analysis. Previous reports indicate that few genes in the *Gonium* and *Volvox* genomes show a signature of undergoing positive selection, as measured by the ratio of non-synonymous to synonymous mutations (*d*N/*d*S)^8^. Using *Chlamydomonas* as the reference for ratio calculation 77, 51, 4, 19 and 3 genes exhibited positive selection in *Gonium, Pandorina, Yamagishiella, Eudorina*, and *Volvox,* respectively (Fig. 2G). Using *Volvox* as the reference, there were 372, 82, 53, 4 and 17 genes with a significant *d*N/*d*S value in *Chlamydomonas*, *Gonium*, *Pandorina*, *Yamagishiella,* and *Eudorina*, respectively (Fig. 2G). Among multicellular volvocines, *Gonium* has a larger number of genes under positive selection, with the multicellular species examined having comparable albeit lower numbers of genes under positive selection. These results suggest that positive selection is not the only force involved in volvocine evolution; rather, it seems like a combination of processes (co-option, gain, and loss of genes) might have set the stage for instances of positive selection, especially upon the transition to undifferentiated multicellularity.

### Gene loss occurs mainly by progressive decay

As means to distinguish between evolutionary gene loss events (and the mechanism underlying them) and false positive losses from technical limitations, we examined conservation of genome structure at the sequence level for all volvocine genomes. This approach is difficult to do in other phyla, where gene loss is inferred indirectly through phylogenetic analysis^16,17^. The genomes of the volvocines, however, are highly syntenic^8,26^. The remarkable conservation of syntenic regions across volvocine genomes allows for detailed examination of gene loss events, as syntenic blocks of conserved genes may have remnants of genes lost. Cross-syntenic block comparisons between species were made against the best annotated genome for the least developmentally complex organism available (*Chlamydomonas*). *Chlamydomonas* genes from contracting orthologous groups that are not assembled into chromosomes were filtered out. If syntenic regions could not be found for the selected *Chlamydomonas* genes and their orthologs in the other five species, those genes would too be eliminated from further analysis. Thus, only a sub-set of conserved loci where gene order was preserved in well-assembled, well understood and syntenic sequence blocks that belong to contracting orthologous groups was examined.

**Figure 3:**
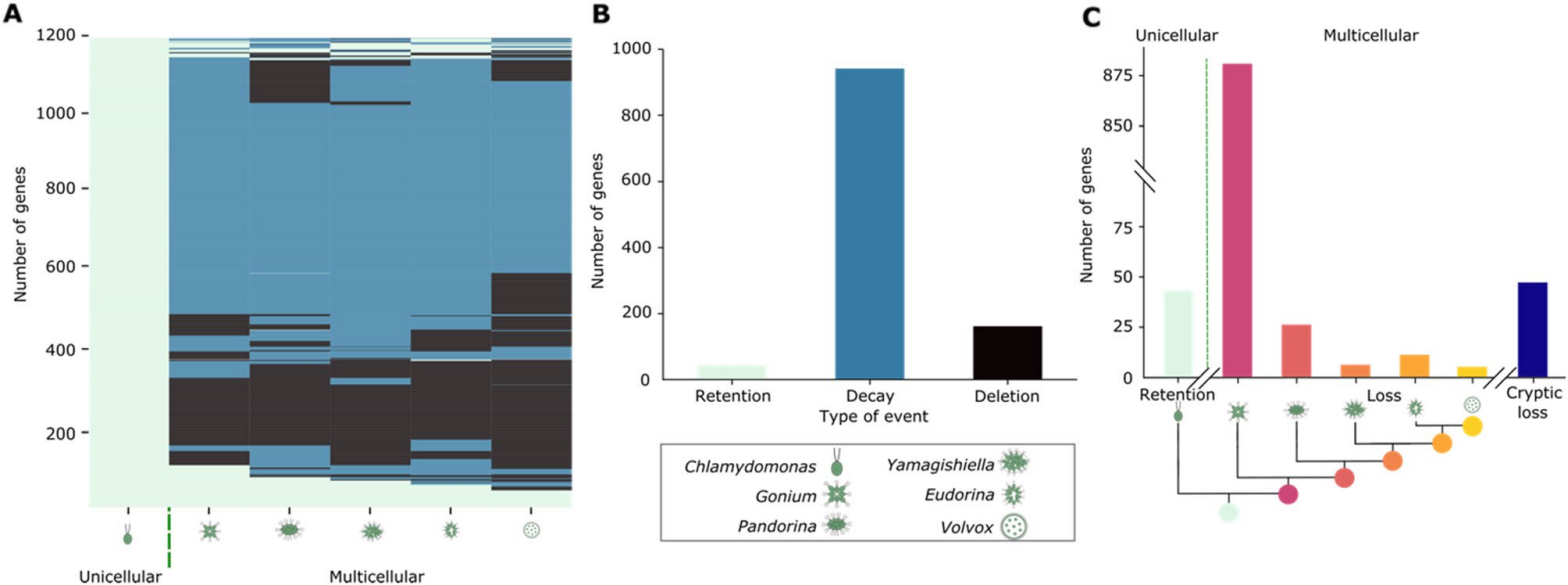
Gene loss in the volvocine algae occurs primarily by gradual decay. (**A**) Retention (light green), decay (blue), and deletion (black) of 1184 loci belonging to orthologous groups undergoing contraction in volvocine species. (**B**) Cross species quantification of retention (light green), decay (blue), and deletion (black) of loci in contracting orthologous groups. (**C**) Quantification of events of retention and loss by decay relative to evolutionary relationships between volvocine species. Losses that do not follow phylogenetic distributions are considered cryptic (dark blue).

Examination of the mechanisms underlying gene loss was performed on genes belonging to the 226 contracting orthologous groups at the sequence level (Fig. 2A). The contracting groups contain 1230 genes assembled into *Chlamydomonas* chromosomes, of which 1184 (91.2%) in 199 (88.1%) orthologous groups had loci that were adequately assembled and syntenous in all genomes for further analysis. This analysis demonstrated 43 of 1184 genes in contracting orthologous groups are retained—that is, that despite belonging to contracting orthologous groups, these genes have an ortholog in all six volvocine species, while 1,141 loci exhibit gene loss (Fig. 3A,B). The mechanisms of gene loss were then examined by tBLASTx to determine if remnants of the lost genes (gene decay) were present at the locus or if remnants of the gene were completely lost (gene deletion). Our results show gene decay is more abundant than deletion in all the colonial species; 72.7% of *Gonium,* 65.8% of *Pandorina*, 72.9% of *Yamagishiella*, 73.2% of *Eudorina* and 51.4% of *Volvox* gene losses show decay signatures (Fig. 3A). Observed signatures of decay confirm that our observations of gene loss are not likely to be caused by technical issues with genome comparisons, but rather are, for the most part, evolutionary losses of gene function. Thus, the volvocines exhibit a burst of gene loss primarily by decay during the transition to undifferentiated multicellualrity that appears to have continued as developmental complexity increased.

Cross-species comparisons of retention, decay, and deletion events show that most candidate gene losses, 975 loci (82.3%), are due to decay (Fig. 3B). Surprisingly, our analysis also suggests 1,017 (85.9%) loci are gene loss candidates in all five multicellular species. Of these, 880 (74.3%) occurred by gradual decay (Fig. 3C, Supplementary Table S8). The presence of decay signatures eliminates the possibility that these loci represent gene gains of *Chlamydomonas*. In the case of deletions, only 166 loci (14.0%) were completely absent in all multicellular species, which could either represent complete gene loss in the multicellular lineage or *Chlamydomonas-*specific gene gains. GO-term analysis of *Chlamydomonas* orthologs of retained, decayed, and deleted genes was performed. Since the GO annotation of volvocine genomes is far from complete, only 24 of 43 retained gene orthologs, 234 of 975 decayed gene orthologs, and 42 of 166 lost gene orthologs could be analyzed. Genes associated to conserved biological processes (e.g. metabolic processes, ion transport) and molecular functions (e.g. protein kinase activity, nucleic acid binding) have undergone loss by decay, deletion, or both (Supplementary Fig. S4). The predominance and progressive distribution of decay signatures suggest that a major gene loss event(s) occurred after the LCA of colonial volvocine algae diverged from the lineage of rather than that the *Chlamydomonas* lineage experienced extensive gene gains after diverging from multicellular volvocines.

### Neither co-option nor gene age account for the all of volvocine developmental complexity

Previous analysis of the volvocine genomes indicated that co-option of genes has occurred in *Gonium* and *Volvox*^8^. In this expanded phylostratigraphic analysis we confirmed the high degree of conservation of ancestral genes between volvocine genomes, as 51.1 to 69.8% of the genes per species fell under phylostratum 1 (PS-1, cellular organisms) and PS-2 (Eukaryotes) (Supplementary Fig. S5). Multicellular volvocines are traditionally divided into three families (Tetrabaenaceae, Goniaceae, and Volvocaceae); this means that genetic innovation relating to increased developmental complexity should be represented in family and genus strata. Contrary to that expectation, 0 to 4.3% of the genes per species were found in PS-7 (family), while 0 to 9.1% of the genes per species were found in PS-8 (genus). These results support co-option as a key mechanism in volvocine evolution, and do not coincide with a scenario of increases in developmental complexity resulting from matching genetic innovation, as previously thought^6^.

Furthermore, genes undergoing decay and deletion are not exclusively younger, dynamic genes. *Chlamydomonas* orthologs of decaying genes are conserved; 72.2% fall under phylostrata 1 to 6 (Supplementary Fig. S6). Likewise, 73.7% of the *Chlamydomonas* orthologs for deleted genes are classified into PS1-6 (Supplementary Fig. S6). These results suggest that while roughly 27% of observed gene loss events reflect the evolutionary dynamics of young genes that might not be involved in increasing developmental complexity, most of the observed losses are likely linked to more consequential evolutionary events.

Previously, genes important for *Volvox* development were hypothesized to reflect key co-option events, including those needed for the evolution of multicellularity^3,8,26^. Hence, phylogenetic analyses were used to gain deeper understanding of co-option in the volvocine algae. Cyclin D1 genes (Supplementary Fig. S7F), certain extracellular matrix (ECM) related genes (Supplementary Table S9, Supplementary Figs. S8, S9), and the *Volvox* cell differentiation gene *regA*^34^ follow a duplication and divergence pattern that coincides with increases in complexity. However, other developmentally relevant genes such as other cyclins (Supplementary Fig. S7A–E), *invA, B, C,* (Supplementary Fig. S10) and *glsA,* (Supplementary Fig. S11) do not. Genes that encode intrinsically disordered proteins (IDPs) were also examined, since these proteins might be co-opted for novel developmental functions in the volvocine algae^35,36^. A relationship between complexity and protein disorder in TFs has been reported for a variety of organisms^37^. However, no significant change in the IDP content, frequency or distribution was observed between the volvocine algae genomes (Supplementary Fig. S12), even when testing exclusively for genes encoding protein-binding proteins (Supplementary Fig. S12A).

### Protein–protein interactions differ between volvocines even for conserved proteins

**Figure 4:**
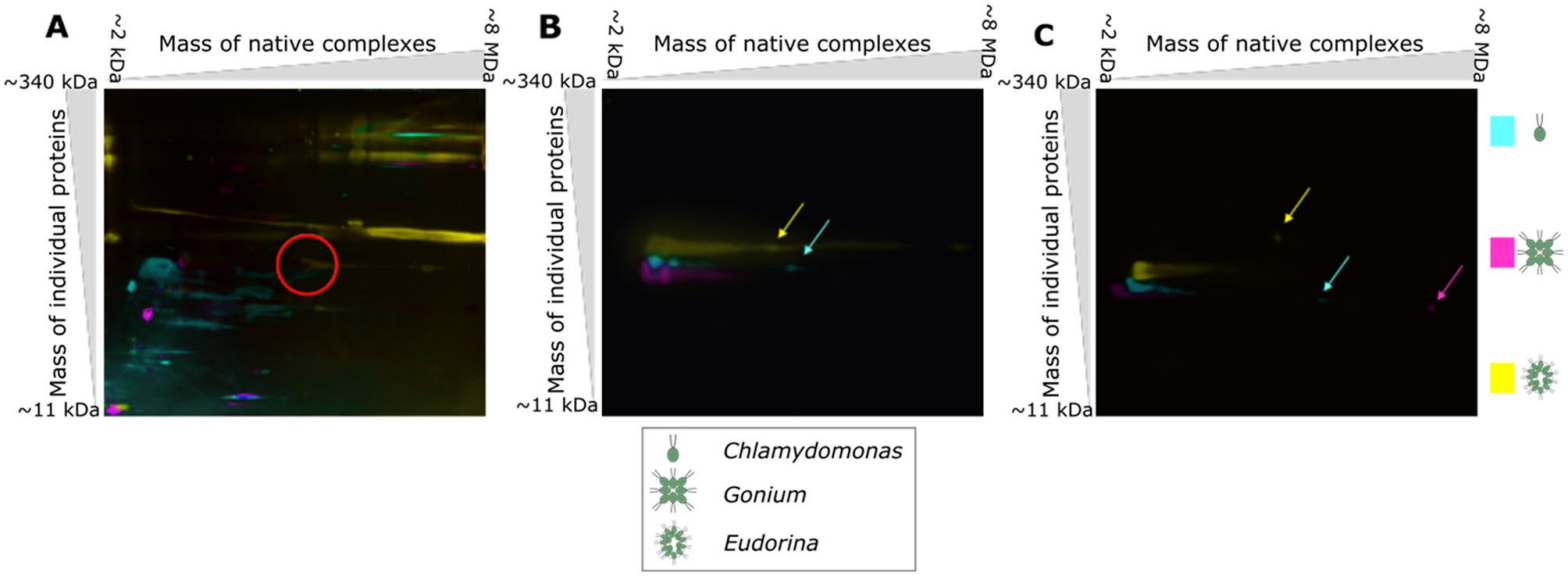
Protein–protein interactions differ among volvocine species. Blue Native (BN) PAGE followed by SDS-PAGE of lysates from *Chlamydomonas* (pseudocolored cyan)*, Gonium* (pseudocolored magenta)*,* and *Eudorina* (pseudocolored yellow) were performed. (**A**) Overlay of silver stains. RuBisCO signal is circled in red. (**B**) Overlay of western blot signals for α-tubulin. Tubulin signals for species-specific complexes are indicated by color coded arrows. (**C**) Overlay of western blot signals for β-actin. Actin signals from unique complexes are indicated by color coded arrows.

To understand how developmental complexity could evolve in the context of gene loss in the volvocine algae, the dynamics of their protein–protein interactions (PPIs) were examined. We reasoned that changes in PPIs could lead to impactful consequences to volvocine developmental complexity, even though few genes are undergoing positive selection or co-option (Fig. 2G). Examination of silver-stained 2D-PAGE gels of total protein extracted from *Chlamydomonas, Gonium* and *Eudorina* shows extensive differences in the proteomic makeup of each species (Fig. 4A, Supplementary Fig. S13A).

Changes in morphological and developmental complexity in the volvocine algae are thought to be related to the cytoskeleton^38^. This is because cytoskeletal elements common to *Chlamydomonas* and *Volvox* are involved in cell division in both species, and in the latter also in inversion^38^. However, many cytoskeletal proteins are also highly conserved, as they are components of structures with essential roles in the cell^39^. Tubulin and actin proteins are highly conserved in the volvocine algae (Supplementary Fig. S14). Hence, the PPIs of actin and tubulin were examined to test for protein-level functional diversification in the context of changes to developmental complexity. As expected, complexes of similar size were found in *Chlamydomonas, Gonium,* and *Eudorina* 2D-PAGE immunoblots (Fig. 4B,C, Supplementary Fig. S13), where minor differences in migration patterns for both proteins in the denaturing dimension are likely caused by post-translational modifications. However, novel α-tubulin complexes were shifted along the native axis, suggesting the presence of larger, species-specific complexes containing tubulin (Fig. 4B, arrows). Similarly, 2D-PAGE immunoblots for β-actin showed a common streak in all three species, but also displayed species-specific actin-containing complexes that are larger than the shared complex (Fig. 4C, arrows). Cultures used to extract protein were asynchronous; hence, the differential migration patterns between species are not likely to result from comparing species at differential life stages. Taken together, these results indicate that while the genomes of the volvocine algae are highly similar and experiencing gene loss, PPIs between conserved gene products are forming species-specific complexes, which could account for the observed differences in biological complexity between the volvocines.

### Gene loss is a driver for regulatory innovation

**Figure 5:**
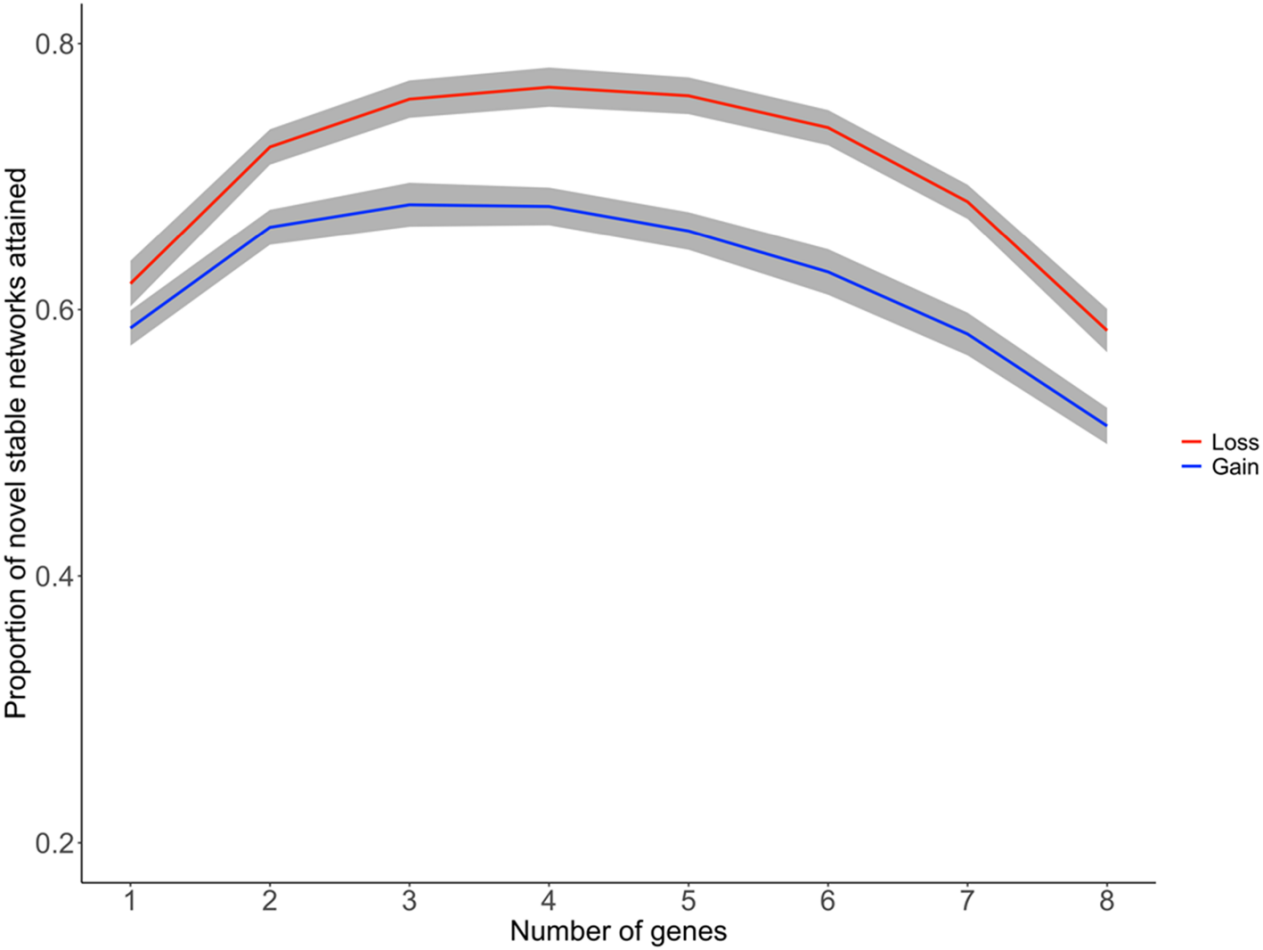
Gene loss in a fully connected network (*c* = 1) of genes (*N* = 10) yields a higher proportion of novel stable network states than gene gain. Wagner model simulates loss (red) and duplication (blue) of *k* genes. Grey shading represents variance.

To evaluate the potential of gene loss as a mechanism of evolutionary transitions, gene loss and gain were modeled using an adaptation of the Wagner gene regulatory network model^40^. Our model mirrors the effects of gene gain in Wagner’s original model, which demonstrated that networks more often reach a new equilibrium state following an intermediate number of gene duplications than when there are many or few duplications^40^. Our model predicts similar outcomes following gene loss with the proportion of novel stable gene network configurations being higher following gene deletions as compared to an equal number of duplications (Fig. 5). Upon iterating the model over a broad range of network connectivity, similar results were obtained (Supplementary Fig. S15). The effect of gene loss was also simulated with random networks that mimic the topology of empirically observed gene interaction networks. We see similar results for intermediate levels of gene deletion, with even greater effect on the remaining genes when most genes are deleted (Supplementary Fig. S16). Through network-wide loss of robustness, novel stable network states might result in regulatory rewiring that allows for major changes to developmental programs. In sum, our evidence indicates that the signature of gene loss in the volvocine genomes may represent an example of a broadly used mechanism for the evolution of developmental complexity.

## Discussion

Our work further challenges the common assumption that developmental complexity and genomic repertoire are positively correlated^41,42^. If this were the case, the developmental gains in *Volvox* and species that exhibit seemingly intermediate steps of developmental complexity (typified by *Gonium, Pandorina, Yamagishiella* and *Eudorina*) might be the consequence of the acquisition of novel genes and/or the result of co-option events correlating to their developmental gains^3,34,43,44^. However, we discovered that a very small set of expanding and co-opted genes are found in the five multicellular species (Supplementary Table S9, Supplementary Figs. S7–S11), some of which have an evolutionary signature of sequential co-option. With few exceptions (e.g., *reg,* extracellular matrix (ECM) genes, cyclin D1), it was not possible to assign novel genes to specific developmental gains in the volvocine algae, despite the complexity of these novel traits.

Notwithstanding the similarities between volvocine genomes (Fig. 1B,C), genetic losses in conserved genomic regions have occurred in volvocine history (Fig. 3). Orthologous groups and Pfam domains both show more numerous significant gene losses than they do gains, and the rate of loss for some of these groups can be quite dramatic (Fig. 2A,B), even in the absence of significant variation in the number of Pfam domain counts per gene between the six species (Supplementary Fig. S1). These results agree with previous work describing pervasive Pfam loss events across eukaryotes^45^. Regulatory and histone genes, which are important for development^8,32,46^, are not exceptions to this trend (Fig. 2C,D,E). The progressive contraction of histone genes as complexity increases for the volvocine lineage allows for the possibility of gene loss to be directly related to increased complexity in this lineage, either as a driver or as a consequence of shifts in selective pressures.

The non-uniform distribution of gene loss events across the five multicellular species (Fig. 3A,C), as well as the signatures of decay found for many of these genes (Fig. 3B), contend that the observed patterns are not a consequence of annotation disparities or analytical biases. Events of gene loss throughout evolution are not unique to the volvocines, suggesting that this is a widespread phenomenon^21–23^. However, this is the first time to our knowledge that the mode of gene loss has been traced within a lineage. The mechanisms underlying the observed rates of decay for genes lost remain enigmatic, but their presence allows for the inference that most gene loss events described here are *bona fide* and not *Chlamydomonas* specific gene gains. Evidence for gene loss through gradual decay in the context of minimal duplication and divergence differs from the model of differential loss of paralogs that has been proposed for metazoans^16^. It also appears to differ from other lineages^45^ in the sense that there is no clear functional bias related to gene loss (Supplementary Fig. S4); instead, it would seem that a wide array of functions are differentially preserved.

Previous reports posited that gene duplication is a costly process due to the expense of synthesizing the gene products and the impact of increased function/gene dosage alteration on the cell^47^. Moreover, novel stable regulatory networks can be readily generated via gene loss (Fig. 5, Supplementary Fig. S15). Hence, both theoretical and biological systems suggest that gene gains are neither the only nor the fastest route to biological innovation. Indeed, it has been hypothesized that the addition and subtraction of elements in regulatory networks across species^48^, as well as co-option and differential spatiotemporal combinations of conserved genes/networks and processes^49^ participate of generating some key aspects of organismal body plans. Our data supports the idea that changes to developmental complexity, such as the transition to multicellularity, could be driven by re-purposing existing genes for new functions while eliminating others as opposed to by generating of extensive genetic novelty^50^.

The patterns of gene loss and PPI diversification in the volvocine algae illustrate vigorous genomic and proteomic shifts that likely may underlie changes to evolutionary and developmental constraints between members of a single lineage. A significant proportion of the gene losses (~ 55%) identified in the volvocine algae were shared by the five colonial species, whereas other gene loss patterns were less numerous (Fig. 3C). The ancestor of undifferentiated *Gonium* (and the other multicellular species studied) would then appear to be a candidate hotspot for initiation of gene loss and co-option. Analysis of genes with a signature for positive selection shows *Gonium* has the largest number of genes under positive selection among the species we studied (Fig. 2G), and it appears to have undergone diversification of histone H3 N-terminal tails (Fig. 2F). Perhaps the developmental complexity portrayed by *Volvox* was made possible by the same mechanisms and tools visible in *Gonium*, such as co-option of RB^3^*.* Recent findings suggest volvocine undifferentiated multicellularity has evolved more than once^51^. Likewise, differentiated multicellularity has arisen multiple times^13,52^, as has the volvocine spheroidal colony morphology^53^. For instance, *Astrephomene* is notably similar to *Eudorina* phenotypically despite not belonging to Volvocaceae^51^, and its embryogenesis and development is markedly different from that of other spheroidal volvocines^53^. Future work comparing independent instances of evolution of simple multicellularity will no doubt provide valuable insight into the mechanistic similarities and differences underlying these convergent phenotypes. For the time being, the comparisons between *Chlamydomonas, Gonium, Pandorina, Yamagishiella, Eudorina* and *Volvox* support the possibility that complex multicellularity is a relatively straightforward consequence of simple multicellularity. Should this be the case, a future challenge would be to test this hypothesis in other multicellularity models as means to assess if the true evolutionary transition lies in establishing the conditions that stabilize undifferentiated multicellularity through loss of gene functions that might be necessary to maintain the unicellular state. In sum, our results indicate that biological innovation coincides with gene loss events, which could allow for reconfiguration of gene networks, differential use of existing functional repertoires, and reduction of the impact of genetic gains.

## Methods

### Strains and genomes

*Chlamydomonas reinhardtii* CC-503 MT^+^ genome version 5.5^11^ (GCA_000002595.3), *Tetrabaena socialis* NIES 571 (GCA_002891735.1)^54^, *Gonium pectorale* K3-F3-4 MT^−^ NIES-2863^8^ (GCA_001584585.1), and *Volvox carteri f. nagariensis,* Eve S1^26^ genome versions 1 (GCA_000143455.1) and 2.1 (available on Phytozome)^26,55^ were used for all analyses. Genome assemblies for *Yamagishiella* and *Eudorina* were kindly provided by Dr. Hisayoshi Nozaki before publication^56^. All analyses involving *Yamagishiella* were performed using *Y*. *unicocca* strains 2012-1026-YU-F2-6 MT^+^ NIES-3982, and 2012-1026-YU-F2-1 MT^−^ NIES-3983. For *Eudorina*, *Eudorina* sp. strains 2010-623-F1-E4 female NIES-3984 and 2010-623-F1-E2 male NIES-3985 were utilized. Analyzed red algae genomes have been published previously: *Porphyra umbilicalis*^57^ (GCA_002049455.2), *Pyropia yezoensis*^58^ (GCA_009829735.1), *Chondrus crispus*^59^ (GCA_000350225.2), *Porphyridium purpureum*^58^ (GCA_000397085.1) and *Cyanidioschyzon merolae*^59^ (GCA_000091205.1). Chlorophyte outgroup species genomes have been published previously: *Chromochloris zofingiensis* (available on Phytozome)^60^, *Chlorella variabilis*^61^ (ADIC00000000), *Micromonas pusilla*^62^ (GCA_000151265.1), and *Ostreococcus lucimarinus*^63^ (GCA_000092065.1).

### Algae growth conditions and library preparation

Cultures of *Pandorina morum* 5 (MT^−^), a gift from Dr. Hisayoshi Nozaki, were initially decontaminated by collecting colonies grown in Standard Volvox Media (SVM) by centrifugation at 1000 g for 5 min at 25 °C and removal of the growth media. These colony pellets were overlaid with 50% Percoll^®^ (Millipore-Sigma) in SVM plus Acetate (SVMA) followed by illumination with 100 µE of light at the top of the tube. The top 1 mL of colonies that were able to phototax through this were then plated for single colonies on SVMA agar plates supplemented with 50 µg/mL carbenicillin, 50 µg/mL cefotaxime, 10 µg/mL trimethoprim and 20 µg/mL chloramphenicol. Cultures from single isolates were established in SVM and grown with 0.5% CO_2_ bubbling at 30 °C, 350 µmol m^−1^ s^−1^ light intensity on a 14:10 day:night cycle. Cultures were harvested by centrifugation at 600*g* for 10 min with 0.005% Tween-20. DNA was isolated using a magnetic bead protocol to obtain high molecular weight DNA^64^. Illumina TruSeq PCR-free libraries were prepared from DNA that was either unfractionated or fractionated on a Pippin Prep (Sage Science) in the size ranges of 1 kb, 2 kb, 5 kb and 10 kb. Following library preparation, they were sequenced on an Illumina HiSeq2500 in Rapid Run mode to an estimated coverage of 25X. High molecular weight DNA was also subjected to Pippin Prep fractionation for 15 kb molecules, followed by PacBio SMRTbell library preparation. Libraries were sequenced on a PacBio Sequel system to an estimated 10X coverage. All sequencing was performed by Genewiz (South Plainfield, New Jersey, USA).

*Yamagishiella unicocca* 2012-1026-YU-F2-1 (NIES-3983, MT^−^) and *Eudorina* sp. strain 2010-623-F1-E4 (NIES-3984, female) were axenically cultured for RNA-seq experiments. Algae were grown SVM with 0.5% CO_2_ bubbling at 30 °C, 350 µmol m^−1^ s^−1^ light intensity on a 14:10 day:night cycle. Triplicate cultures were harvested by centrifugation at 600*g* for 10 min with 0.005% Tween-20. RNA was extracted using a Quick-RNA Plant Miniprep Kit (Zymo Research). RNA-Seq libraries were prepared using the TruSeq Stranded mRNA kit (Illumina, Inc. San Diego, California, 20,020,595) according to manufacturer’s conditions.

Axenic cultures for 2D PAGE were grown asynchronously as follows: *Chlamydomonas reinhardtii* strain 21*gr* (*Chlamydomonas* collection CC-4889) was grown in TAP media to a density of ~ 10^6^ cells/mL in ambient conditions with 0.5% CO_2_ bubbling. *Gonium pectorale* (strain Kaneko4, MT^+^ NIES-1711) was grown in SVM + acetate media to a density of ~ 10^6^ colonies/mL in ambient conditions with 0.5% CO_2_ bubbling. *Eudorina* sp. strain 2010–623-F1-E4 (NIES-3984, female) was grown in SVM to a density of ~ 10^6^ colonies/mL at 30 °C with 350 μmol m^−1^ s^−1^ light intensity on a 14:10 day: night cycle with 0.5% CO_2_ bubbling.

### Genome assembly

Illumina reads were assembled de novo with ABySS 2.1.5^65^ using a range of k-mers from 21 to 89, in increments of 4. Unitigs from each of the assemblies were then filtered against a database of known bacterial genomes to remove presumed bacterial endophyte sequences. Remaining unitigs were assembled with ABySS under the same conditions as above. Resulting scaffolds were merged to PacBio reads and assembled using Canu 1.8^66^ (Bioproject PRJNA787305).

### Evidence-based gene prediction

The gene models for *Yamagishiella* and *Eudorina* genomes were generated using RNA-seq data (Bioproject PRJNA787306 and PRJNA787302) and the AUGUSTUS 3.2.3 software package^67^, in a manner similar to gene predictions for *Volvox* and *Gonium*^8,26,68^. AUGUSTUS predicted genes were merged with the genome mapped reads and de-duplicated to remove any splice variants.

### Identification of orthologous groups

Orthologous groups of volvocine algae and red algae genomes were determined using OrthoMCL 2.0.9^69^. The optimum inflation value was determined empirically by testing a variety of values ranging from 1.2 to 4.0 in increments of 0.1 (Supplementary Fig. S17). The inflation value of 1.9 was used for all analyses (see Supplementary File 1).

### Identification of Pfam domains

The diversity and abundance of known Pfam domains in the volvocine algae and red algae genomes were identified using using Pfam Scan^70^ and database version 31. Pfam Scan and associated data collection Perl scripts available at: ftp://ftp.ebi.ac.uk/pub/databases/Pfam/Tools/^30^.

### Analysis of other genomic features

Transcription factors and protein kinases in the annotated volvocine algae genomes were identified using the iTAK version 1.7 program and database^71^. The *Chlamydomonas* gene definitions on GenBank were used to identify histone H1 (Accession #XM_001696120) and quantified (Fig. 2E second panel, Supplementary Table S5). Histones H2A, H2B, H3, H4 (Accession # L41841) genes in the volvocine algae were identified using the reciprocal best BLAST hit method with a e-value cutoff of 1e-5. H2A, H2B, H3 and H4 genes were further filtered using e-values of 1e-10 and quantified (Fig. 2E panels 3–6, Fig. 2F first panel, Supplementary Table S6). The first 30 amino acids from each histone were used for analysis of duplication and presence of predicted post-translational modification sites (lysine methylation) using Methylsight^72^. N-tails per histone per species that had different predicted sites with treshold >=0.5 were considered tail variants and quantified (Fig. 2F second panel, Supplementary Table S6). Histone genes were also identified in the five red algae genomes using gene definitions from the *Chondrus* genome annotation (Supplementary Table S7). Eight internal control genes, Actin, mitochondrial ATP-sythase α subunit (mATP-A), mitochondrial ATP-sythase β subunit mATP-B, α-tubulin, β-tubulin, β-2 tubulin, flagellar dynein heavy chain, chloroplast ferridoxin were identified using OrthoMCL (Supplementary Table S4).

### Identification of gene count trends

Rate of gene number change was calculated by applying a linear regression model on the relationship between gene counts per orthologous group per species and distance of said species from their Last Common Ancestor (LCA, Fig. 1A). Only groups with non-identical gene copy numbers and comprising genes from at least 2 species were used for this analysis. Rate of change values per orthologous group were subjected to quartile analysis to identify outlier groups (Fig. 2A). This same analysis was performed on Pfam domains (Fig. 2B), transcription factors (Fig. 2C), and protein kinases (Fig. 2D) for volvocine species and for red algae species. Regression and quartile analyses were performed using custom Python scripts.

The orthologous groups used for the regression analysis were also subjected to gene gain and loss parsimony-based analysis using GLOOME^31^ web server. Since GLOOME requires patterns of presence/absence of gene families to assess gain and loss trends, orthologous groups where the number of member genes per species are all greater than zero were not included. The contracting orthologous groups from the rate of change analysis described above were then searched against the GLOOME output (Supplementary File 2) and quantified. Contracting orthologous groups that belong to the non-included dataset were also quantified.

### Identification of positively selected genes and whole-genome-based phylogeny

Positively selected genes were identified using the PosiGene pipeline^73^. Two independent PosiGene analyses were performed using *Chlamydomonas* and *Volvox* genome annotations as anchors. Chlorophytes *Chlorella variabilis*, *Micromonas pusilla*, *Ostreococcus lucimarinus* and *Chromochloris zofingiensis* were used as outgroups. Similarly, the red algae genes under positive selection were identified using *Porphyra umbilicalis* as anchor species as it has previously been reported to be closest to the LCA of red algae^74,75^. A gene was considered to be positively selected if the *d*N/*d*S value (HA foreground omega) was greater than 1 and the Bonferroni-corrected *p*-value was less than 0.05. As a part of the workflow, PosiGene generates a phylogenetic tree from the isoform assignments and calculates the distance to LCA (Fig. 1A, Supplementary Fig. S18).

### Characterization of gene loss patterns

To evaluate annotation completeness, BUSCO v4.0.2^27^ was run in protein mode using the Chlorophyta lineage dataset for each volvocine species (Supplementary Table S2).

Orthologous groups showing significant contraction (Fig. 2A) were selected for gene loss analysis based on whether they included *Chlamydomonas* genes. *Chlamydomonas* loci that were not assigned to chromosomes were not analyzed further. Orthologous groups that included *Chlamydomonas* loci with the aforementioned characteristics (target loci) served as anchors for obtention of genes up and downstream, called the ‘syntenic neighborhood’. Orthologs for loci in the syntenic neighborhood, as well as target loci for *Gonium, Pandorina, Yamagishiella, Eudorina* and *Volvox* were retrieved from their respective orthologous groups. Instances of no ortholog found for target loci were subjected to tBLASTx^76^ 2.2.18+ searches using tailored databases based on syntenic neighborhoods for each species. Non-informative (loss of syntenic neighborhoods or no BLAST results) genes were omitted from further analyses. tBLASTx hits were filtered for quality (e-value < 1e−5). Hits passing quality threshold were considered to be evidence of a gene remnant suggestive of loss via decay. Genes with no hits passing quality threshold and conserving synteny are candidate deleted genes. Decayed and deleted genes were assigned to bins corresponding to their pattern of absence (e.g. a gene lost by whichever mechanism only in *Gonium,* but present in the other clades; see Fig. 3C and Supplementary Table S8), or by type of event, where decayed gene clusters consist of orthologs with at least one signature of decay in multicellular species, and deleted gene clusters are those where all losses happen via deletion (Fig. 3B). Percentages of decay and deletion cases were calculated, and a Chi square goodness-of-fit test was applied to determine statistical significance of observed patterns using a uniform distribution as expected probability distribution. All analyses except for tBLASTx search were performed using custom Python scripts. Treemaps for GO analysis of retained, decayed and deleted genes were generated using REVIGO^77^ with default parameters and against *Chlamydomonas reinhardtii* database.

### Identification and analysis of known co-opted genes

Cell cycle genes in *Pandorina*, *Yamagishiella* and *Eudorina* genomes were annotated as previously described^8,26^ using tBLASTn. Transcriptome data and manual curation^8,26^ was used to prepare gene models for *Yamagishiella* and *Eudorina*. Known pherophorins are composed of two pherophorin domains (Pfam DUF3707) that are connected by a variable length hydroxyproline-rich repeat region^78^. All genes containing two or more pherophorin domains were identified as pherophorin genes. Matrix metalloproteases (MMPs) contain a single metalloprotease domain (Peptidase M11, PF05548). Genes containing this Pfam domain were selected for the existence of the [HQ]EXXHXXGXXH motif in the gene model^79^. The *invA, invB, invC* and *glsA* genes were identified in *Pandorina*, *Yamagishiella* and *Eudorina* by the presence of kinesin (PF00225), TPT (PF03151), glycosyl-transferase for dystroclygan (PF13896), and DnaJ (PF00226) domains respectively^43,44^.

### Prediction of lineage specific genes

Phylostratigraphy^80^ was used to predict the lineage of genes in the volvocine algae genomes as described previously^8^. The phylogenetic classes of volvocine algae were defined in the input data as per NCBI taxonomy. All protein sequences were searched against the NCBI non-redundant database from version 2.6+^76^, with an e-value cutoff of 1e−3. Phylostratigraphic classification of each gene ranged from PS-1 to PS-9.

### Identification of protein disorder and protein binding site disorder

Protein disorder was calculated using DISOPRED3^81,82^, that yields percentage disorder in each protein sequence as well as that of protein binding sites. The frequency distribution of percentage protein disorder was calculated and plotted.

### 2D gel electrophoresis and silver stain

Approximately 10^6^ cells/colonies of *Chlamydomonas*, *Gonium,* and *Eudorina* were pelleted and snap frozen in liquid nitrogen. Cells were lysed by six rounds of liquid nitrogen freeze/thaw lysis in in low salt TBS (50 mM Tris, 50 mM NaCl, pH 7.5) and protease/phosphatase inhibitors (Plant Protease Inhibitor Cocktail (Sigma, Burlington, Massachusetts), 1 mM Benzamidine, 1 mM PMSF, 1 mM Na_3_VO_4_, 5 mM NaF, 0.5 mM β-glycerophosphate, 0.1 mM EDTA)^83^. Insoluble material was pelleted by centrifugation at 20,000 g for 10 min at 4 °C. Supernatant was removed and protein content was quantified by Bradford assay (BioRad, Hercules, California)^84^. For blue native PAGE, 20 µg of protein per well was loaded into a 4–12% native PAGE gel (Fisher, Waltham, Massachusetts) with native running buffer (Fisher) and light blue buffer at the cathode (Fisher). Gel was run until the Coomassie dye front had exited the gel. Lanes were excised using a razor and incubated in 1 × Tris-MOPS running buffer (Genscript, Piscataway, New Jersey) with 100 mM DTT and 5 mM sodium bisulfite at 55 °C for 10 min. Gel slice was loaded into a NuPAGE 4–12% Bis–Tris gel (Fisher) and run with Tris-MOPS running buffer with 5 mM sodium bisulfite in the cathode buffer until the blue dye front had exited the gel. Silver staining was carried out using a Silver Staining Kit (Pierce) according to manufacturer’s protocols.

### Western blot and antibodies

Western transfer was performed in Tris–Glycine transfer buffer to a nitrocellulose membrane (0.45 µm pore, Pall Scientific, Port Washington, New York)^83^. Membranes were blocked in 1% milk (α-tubulin) or 5% BSA (β-Actin) for 1 h. Primary antibodies (mouse anti-α tubulin, Sigma T6074 1:5000; mouse anti-β actin HRP, Santa Cruz sc-47778 HRP, 1:5000, Dallas, Texas) were incubated at 4 °C overnight with gentle shaking. Membranes were washed with TBS 0.1% Tween-20 (TBST) and tubulin blots were probed with anti-mouse secondary (Pierce 31,430, Rockford, Illinois) for 1 h. Actin blots did not require a secondary antibody, as mouse anti-β actin is already HRP conjugated. Membranes were developed using Premium Western Blotting Reagent (LI-COR, Lincoln, Nebraska) and scanned for chemiluminescence on a C-Digit Scanner (LI-COR).

### Phylogenetic analysis

In order to determine phylogenetic relationships, the genes from all six volvocine genomes and *Coccomyxa subellipsoidea* (Chlorophyta, GCF_000258705.1) were aligned using MUSCLE v3.8.425^85^ and a phylogenetic tree was produced using RaxML v8^86^ with protein gamma model and automatic model selection. A rapid bootstrapping analysis with 1000 bootstrap replicates was performed and a majority ruled consensus tree was produced.

### Regulatory network model

A Wagner regulatory network model^40^ was designed as follows: a gene network of *N* genes whose on or off expression state at a time $t$ is given as:

$$\vec{S}\left( t \right): = \left( {S_{1} \left( t \right), \ldots ,S_{N} \left( t \right)} \right)$$

where ${S}_{i}(t)$ is the expression state of the *i*th gene at time $t$, *S*_*i*_ = -1 reflects an off state, and *S*_*i*_ = 1 reflects an on state. The state ${S}_{i}(t)$ is determined by the network of interactions affecting gene *i*. The expression state of gene $i$ can be changed due to regulatory interactions. These changes can be modelled as a set of difference equations:

$$S_{i} \left( {t + \tau } \right) = \sigma \left[ {\mathop \sum \limits_{j = 1}^{N} w_{ij} S_{j} \left( t \right)} \right]$$

Here, $\sigma (x)$ is the sign function similar to Wagner’s model and ${w}_{ij}$ is the weight of interaction between gene *i* and gene *j*. The connectivity matrix is given by $w$. Overall connectivity of the network ($c$) is given by the average fraction of entries in $w$ that are different from zeros (i.e. $c\in (\mathrm{0,1})$). The effect of gene duplication on a random network was assessed as in Wagner^40^.

The effect of gene deletion on a random network was assessed as follows. A network of size *N* ($N=10$) was considered. The initial and final states of the gene network were randomly chosen where each individual gene has an equal probability of being on or off (i.e. $p\left({S}_{i}\left(0\right)=1\right)=p\left({S}_{i}^{eq}=1\right)=0.5$). Entries in $w$ were randomly and independently chosen from the Gaussian distribution ($\mu =0,\sigma =1$). Many randomly generated networks result in stable cycles^87^, but analyses of the Wagner model often consider networks with these expression patterns to be non-viable^40,88–90^. We similarly focus our analysis on connectivity matrices that lead to networks with stable fixed point expression patterns (${\overrightarrow{S}}^{eq}$) as $t\to \infty$. We iterated the difference equation 100 times to confirm if $w$ results in ${\overrightarrow{S}}^{eq}$ when the initial state is $\overrightarrow{S}\left(0\right)$. If not, a new $w$ was chosen. Once the network set $(\overrightarrow{S}\left(0\right),w,{\overrightarrow{S}}^{eq})$ was found, *k* genes $(k\in \left(\mathrm{1,9}\right))$ were randomly deleted from the $\overrightarrow{S}\left(0\right)$ to give ${\overrightarrow{S}}^{\Delta }\left(0\right)$. This was done by setting the expression state of the $k$ genes to off state (i.e., − 1). The rows and columns of the connectivity matrix $w$ associated with k genes were set to zero to obtain the updated matrix. The updated connectivity matrix $w$ was then used to simulate the new final expression state ${\overrightarrow{S}}^{\Delta eq}$. Like the Wagner’s gene duplication model, the Hamming distance (${d}_{{h}_{eq}}$) was calculated between ${\overrightarrow{S}}^{\Delta eq}$ and ${\overrightarrow{S}}^{eq}$ assuming it to be proportional to the Hamming distance between the first step simulation (${d}_{{h}_{1}}$) of the original and deleted gene expression states. However, unlike the Wagner’s model, we have state 0 for deleted genes in ${\overrightarrow{S}}^{\Delta eq}$. Because of this, the equilibrium states before and after gene deletion are different in at least k positions. So, we calculated the proportion of instances where ${d}_{h}>k$ to evaluate the effect of gene deletion on the remaining gene expression states. For each $k$, 1000 iterations of simulation were done.

To understand whether network topologies have any effect on our results, we further evaluated the effect of gene deletion on random network topologies that mimic the topologies of real biological networks. For this, we used the SeqNet package in R to generate adjacency matrices for random networks^91^. The adjacency matrix was then scaled by the weight from $w$ to obtain a new connectivity matrix. Everything else remains the same as before where we first search for $\left(\overrightarrow{S}\left(0\right),w,\overrightarrow{{S}^{eq}}\right)$ set and then evaluate the effect of gene deletion. Additionally, to check whether the degree of the network connectivity has any effect on the outcomes of gene deletion, we fixed the number of genes deleted in a network and varied the connectivity from low (0.1) to high (0.9). For each connectivity value, 1000 iterations of simulation were done, and the proportion of instances where ${d}_{h}>k$ were determined. Calculation of proportion of novel stable networks for gene gain and loss was performed using custom scripts.

## Supplementary Information

Supplementary Information 1.

Supplementary Information 2.

## Data Availability

Genomic data that supports the findings of this study has been made available by its respective authors in GenBank with the accession codes listed here: *Chlamydomonas reinhardtii* v5.5 (GCA_000002595.3), *Gonium pectorale* (GCA_001584585.1), *Yamagishiella unicocca* MT^+^ plus (GCA_003116995.1), *Yamagishiella unicocca* MT^−^ (GCA_003117035.1), *Eudorina sp.* female (GCA_003117195.1), *Eudorina sp.* male (GCA_003117095.1), *Volvox carteri f nagariensis* v1 (GCA_000143455.1), *Volvox carteri f nagariensis* v2.1, *Porphyra umbilicalis* (GCA_002049455.2), *Pyropia yezoensis* (GCA_009829735.1), *Chondrus crispus* (GCA_000350225.2), *Porphyridium purpureum* (GCA_000397085.1), *Cyanidioschizon merolae* (GCA_000091205.1), *Chromochlorys zofingiensis*, *Chlorella variabilis* (ADIC00000000), *Micromonas pusilla* (GCA_000151265.1), *Ostreococcus lucimarinus* (GCA_000092065.1), *Cocomyxa subellipsoidea* (GCF_000258705.1). Scaffolds from DNA sequencing will be available upon publication. RNA-Seq data that supports the findings of this study will be available in SRA upon publication. GFF files that support the findings of this study will be available as a Bitbucket repository upon publication. Other supplementary data that supports the findings of this study will be available as a Bitbucket repository upon publication.
