# Supplementary material for: Gene loss during a transition to multicellularity
Source: Sci Rep. 2023 Mar 31;13:5268. doi: 10.1038/s41598-023-29742-2 (PMC10066295; doi:10.1038/s41598-023-29742-2)
Supplement: Supplementary file 1 — Supplementary Information 1. [file 41598_2023_29742_MOESM1_ESM.docx]

Gene loss during a transition to multicellularity

Berenice Jiménez-Marín, Jessica B. Rakijas, Antariksh Tyagi, Aakash Pandey, Erik R. Hanschen, Jaden Anderson, Matthew G. Heffel, Thomas G. Platt, Bradley J.S.C. Olson

Correspondence to: [bjsco@ksu.edu](mailto:xxxxx@xxxx.xxx)

**Supplementary figures and tables**

**Fig. S1 – S17**
**Table S1 – S9**

**Figure S1.** Number of Pfams per gene does not change in the five volvocine algae species.

**Figure S2.** Gene copy numbers of nine internal control genes in the six volvocine species.


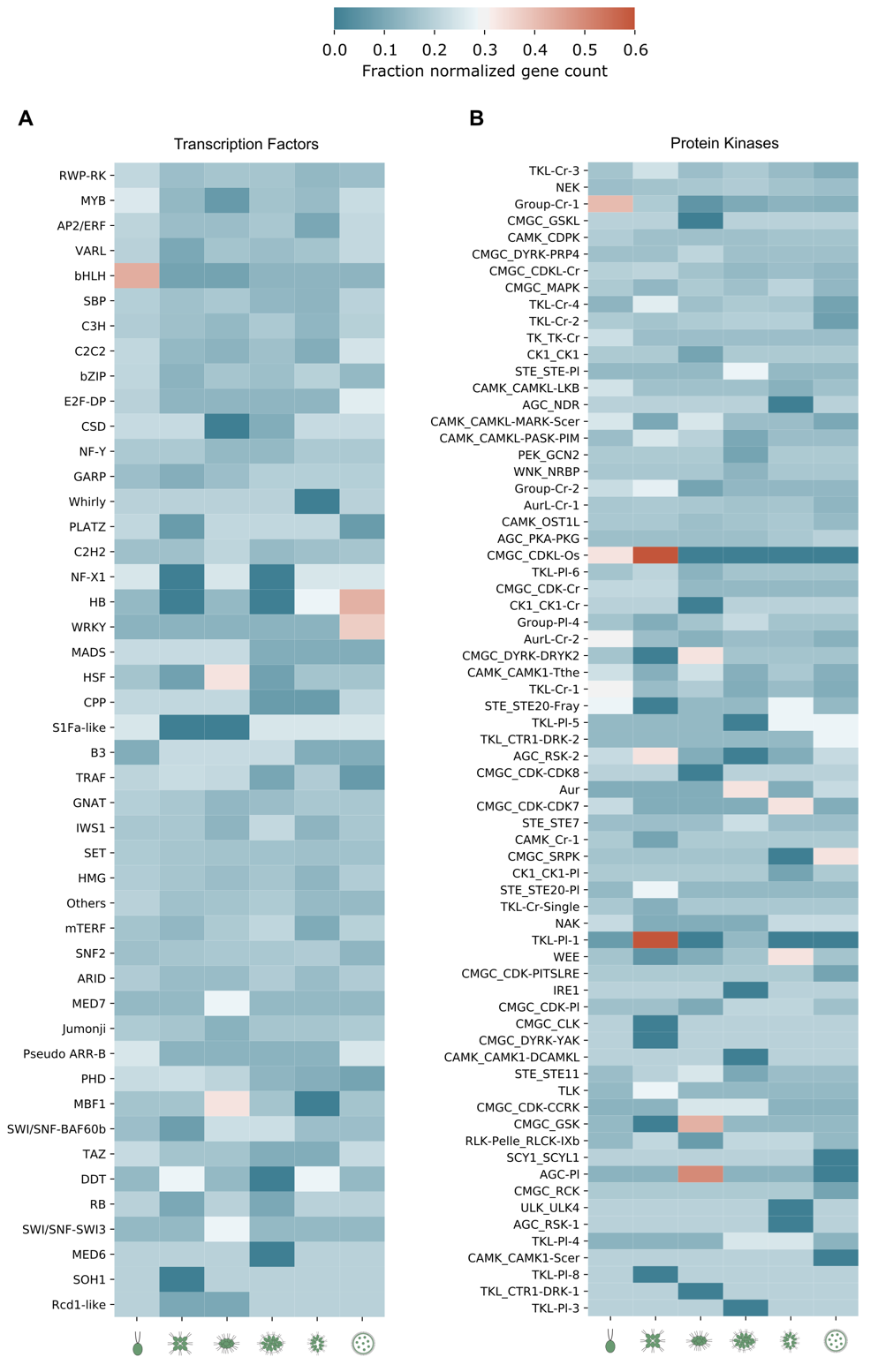


**Figure S3** (A) Transcription factor and (B) protein kinase family loss and gain in the six volvocine genomes. Relative gene counts represent the fraction normalized count relative to counts of the other species.


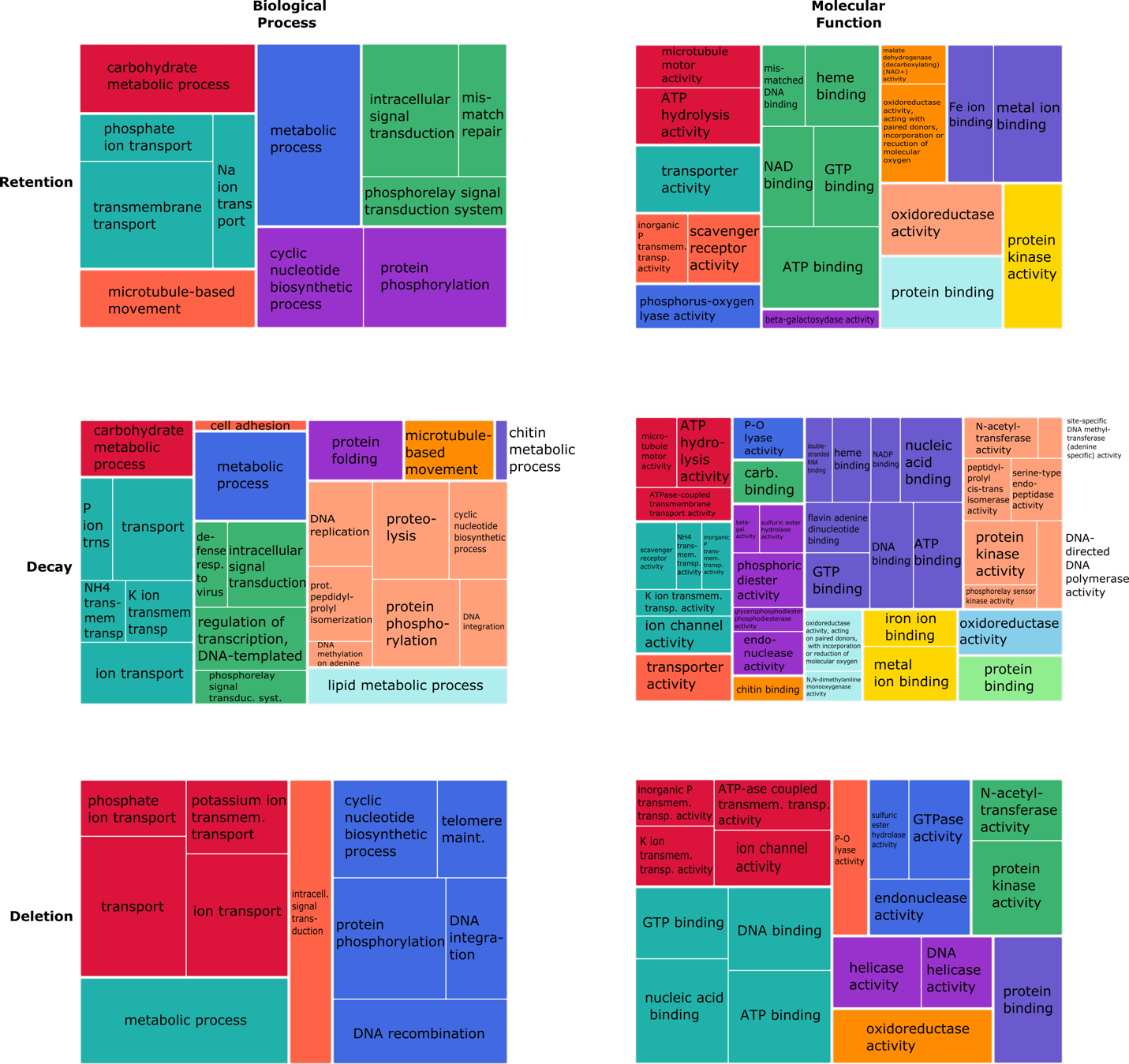


**Figure S4.** GO terms for biological process and molecular functions of *Chlamydomonas* orthologs of retained (55.8% of total retained genes), decayed (24% of total decayed genes), and deleted (25.3% of total deleted genes) genes.

**Figure S5.** Number of genes per phylostratum (PS) for six volvocine species. PS-1 corresponds to cellular organisms (least specific) and PS-9 corresponds to species (most specific).

**Figure S6.** Number of *Chlamydomonas* orthologs of genes that underwent decay or deletion in multicellular volvocines per phylostratum (PS). PS-1 corresponds to cellular organisms (least specific) and PS-9 corresponds to species (most specific).


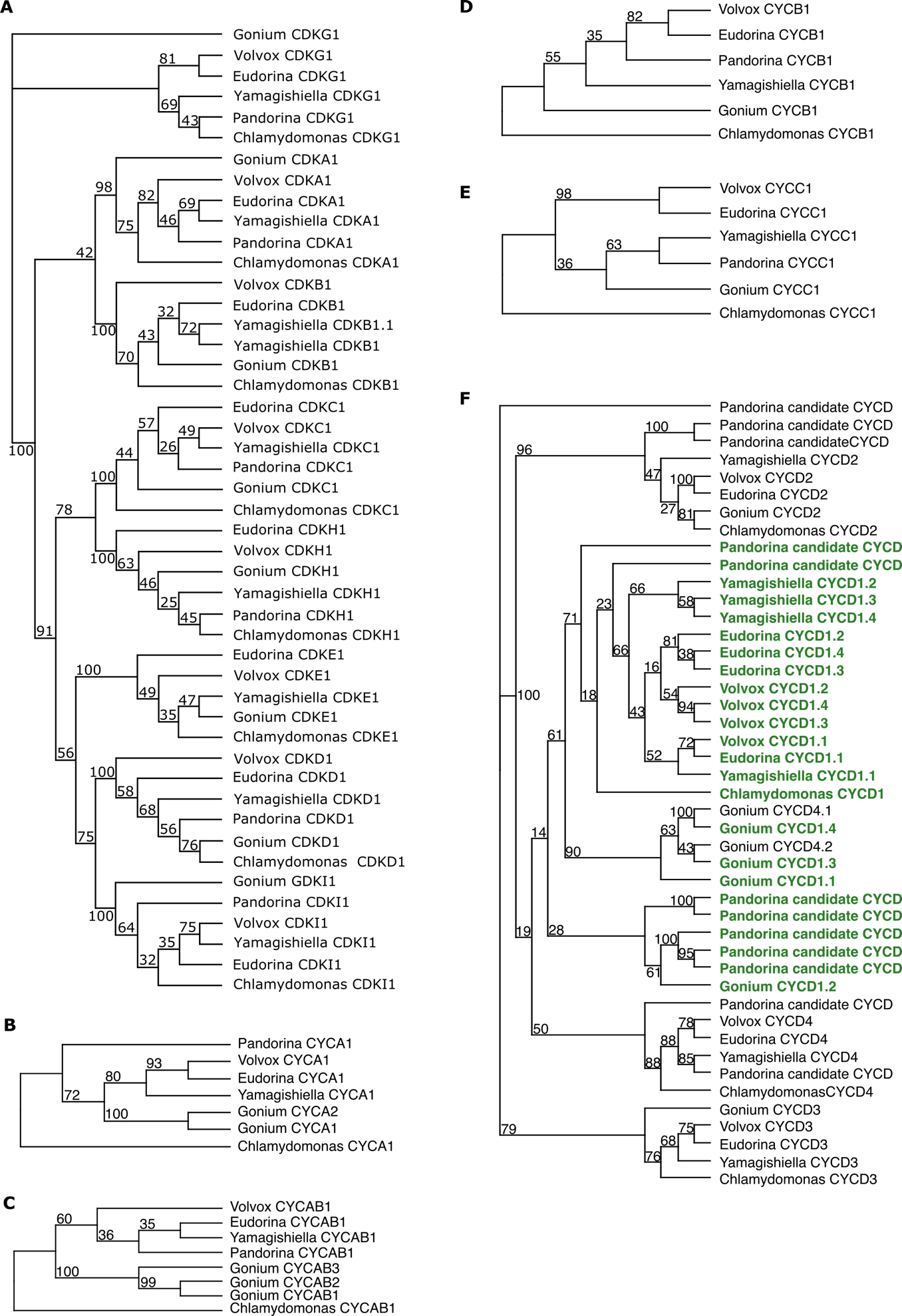


**Figure S7.** Phylogenetic analysis of cell cycle genes**.** (A) Phylogeny of cyclin dependent kinases (CDKs). (B-F) Phylogeny of cyclins (cyc). CycD1s are in green.


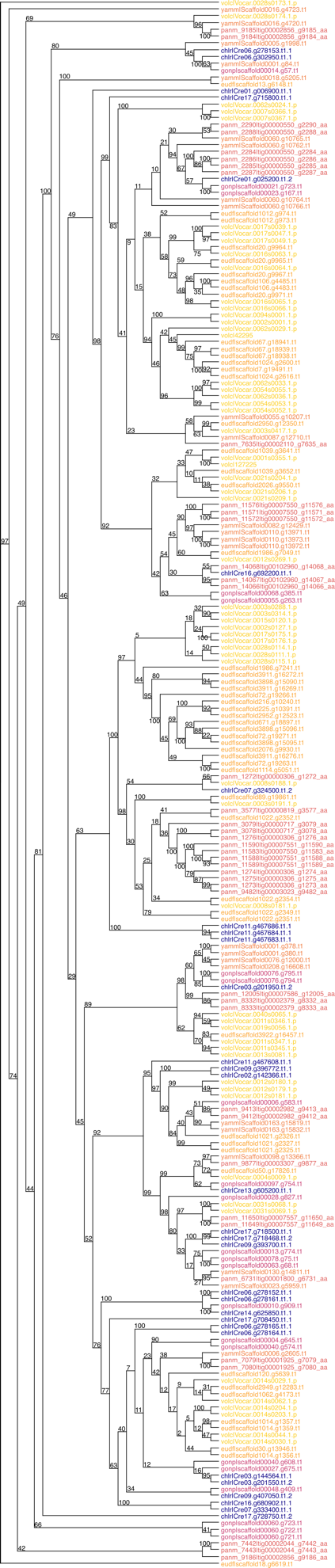


**Figure S8.** Phylogenetic analysis of matrix metalloprotease (MMP) genes. *Chlamydomonas* genes are in blue, *Gonium* in magenta, *Yamagishiella* in salmon, *Eudorina* in orange, and *Volvox* in yellow. Values on the nodes represent percentage bootstrap support.


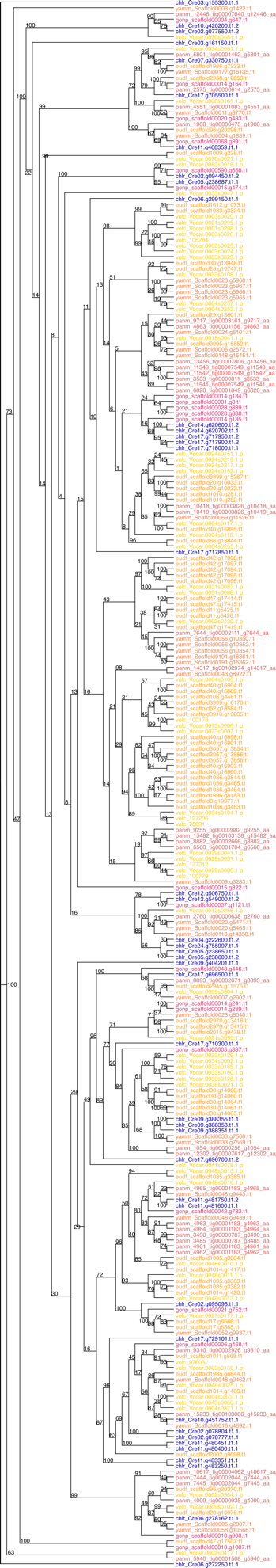


**Figure S9.** Phylogenetic analysis of pherophorin genes. *Chlamydomonas* genes are in blue, *Gonium* in magenta, *Yamagishiella* in salmon, *Eudorina* in orange, and *Volvox* in yellow. Values on the nodes represent percentage bootstrap support.


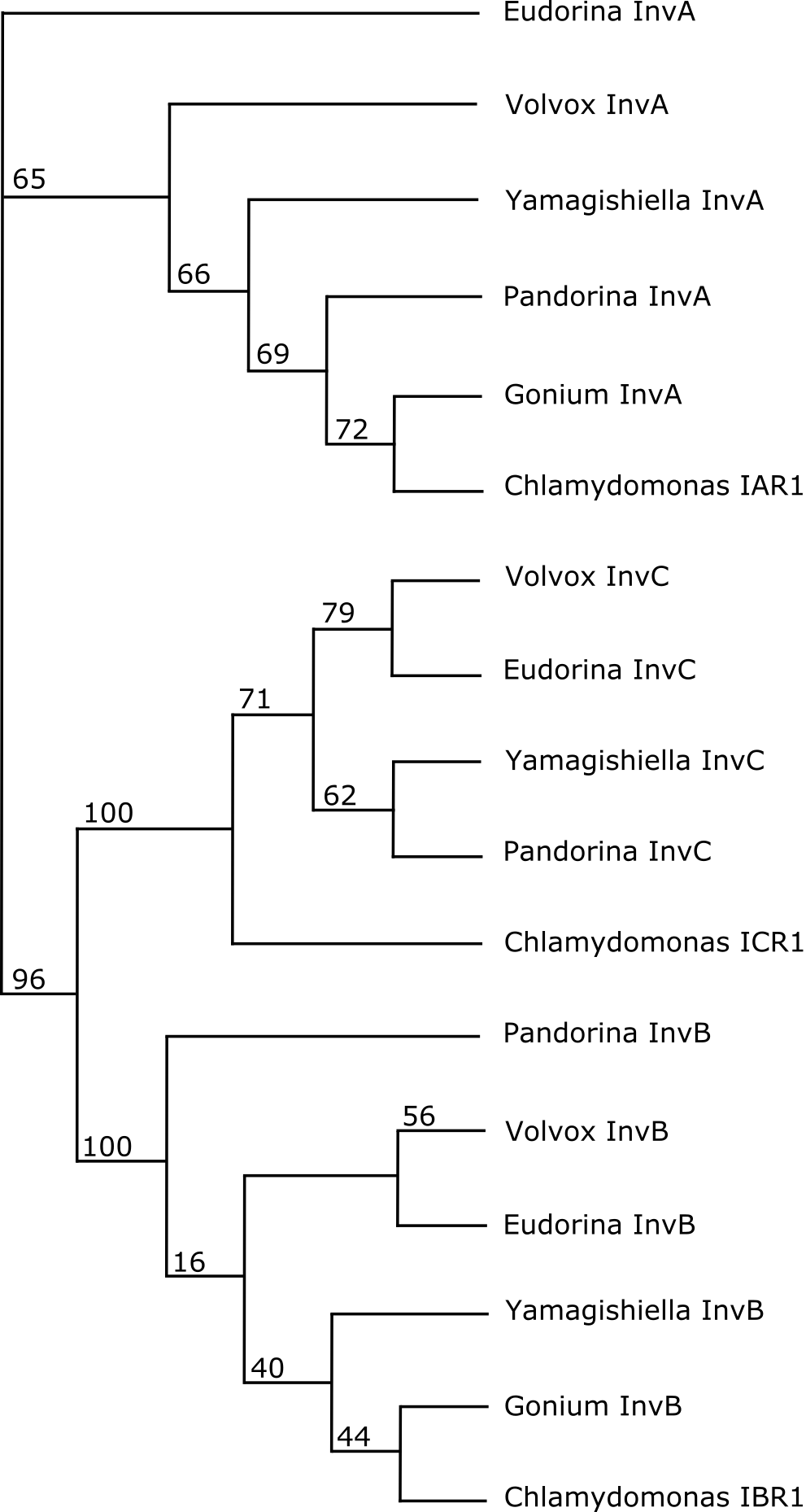


**Figure S10.** Phylogenetic analysis of *inv* genes. Values on the nodes represent percentage bootstrap support.


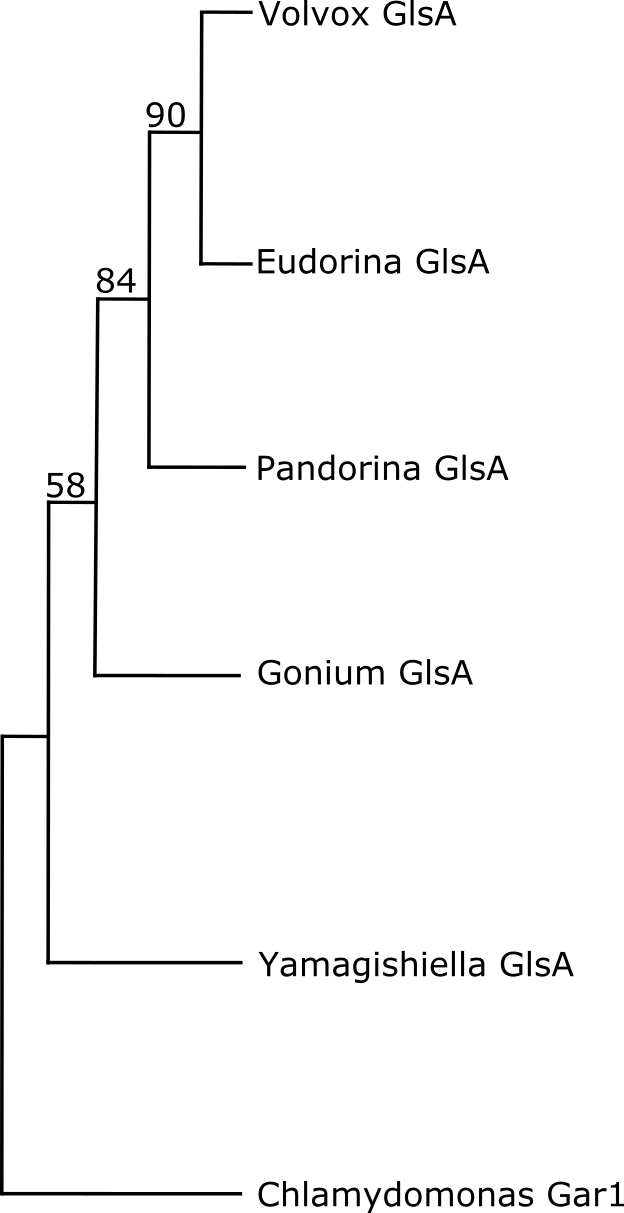


**Figure S11.** Phylogenetic analysis of *glsA* genes. Values on the nodes represent percentage bootstrap support.


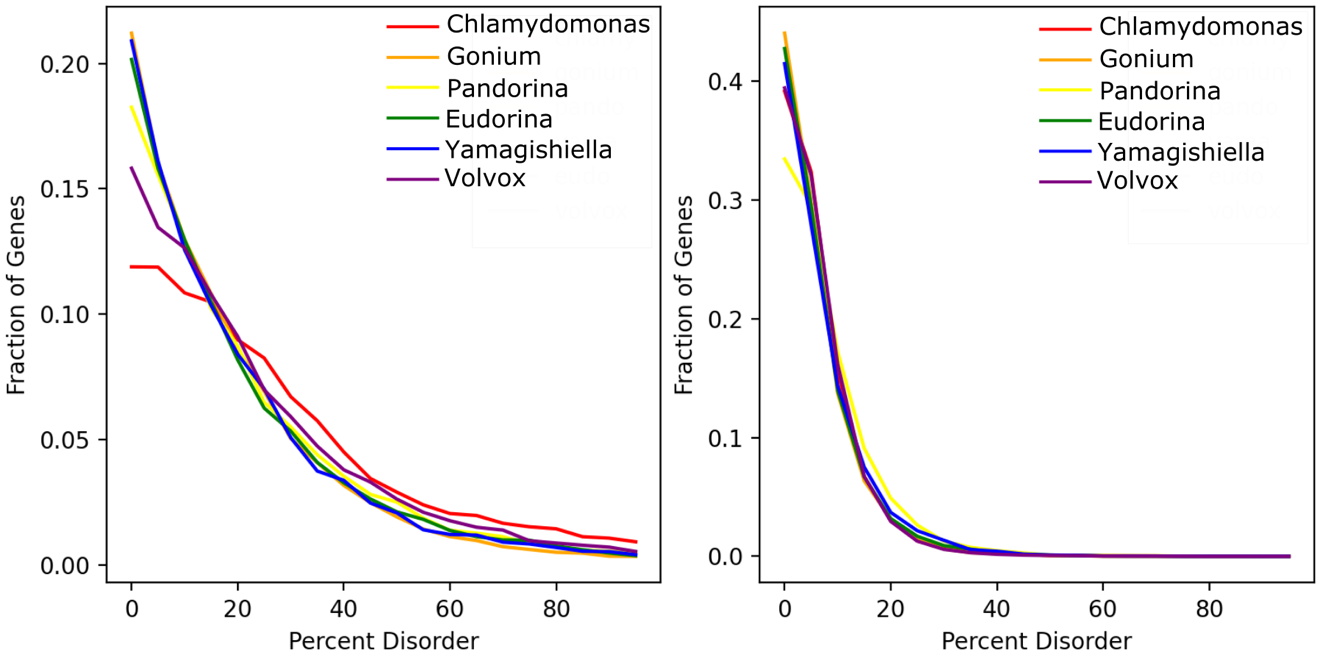


**Figure S12.** Percentage frequency distribution of disordered proteins in the volvocine algae. (A) Percentage distribution of genes encoding protein-binding proteins with an increasing amount of disorder. (B) Percentage distribution of genes encoding proteins with an increasing amount of disorder.


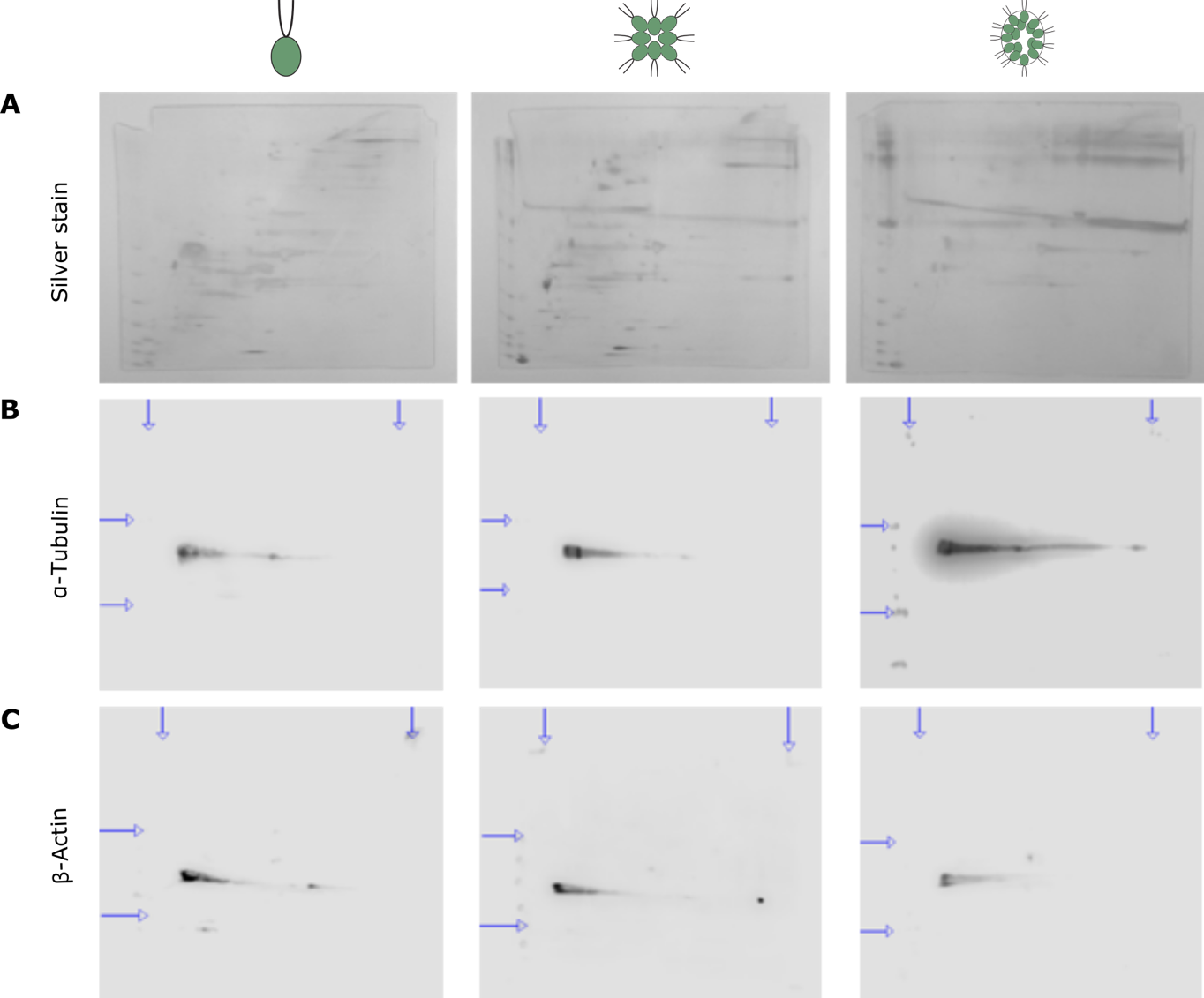


**Figure S13.** Raw images for (A) Silver stained gels; (B) α-tubulin and (C) β-actin blots for *Chlamydomonas, Gonium* and *Eudorina*. Vertical blue arrows indicate boundaries of the native gel slice. Horizontal blue arrows indicate 80 kDa (top) and 25 kDa (bottom) bands on the SDS-PAGE standards ladder. Silver stains are notched at the top corners to indicate the boundaries of the native gel slice.


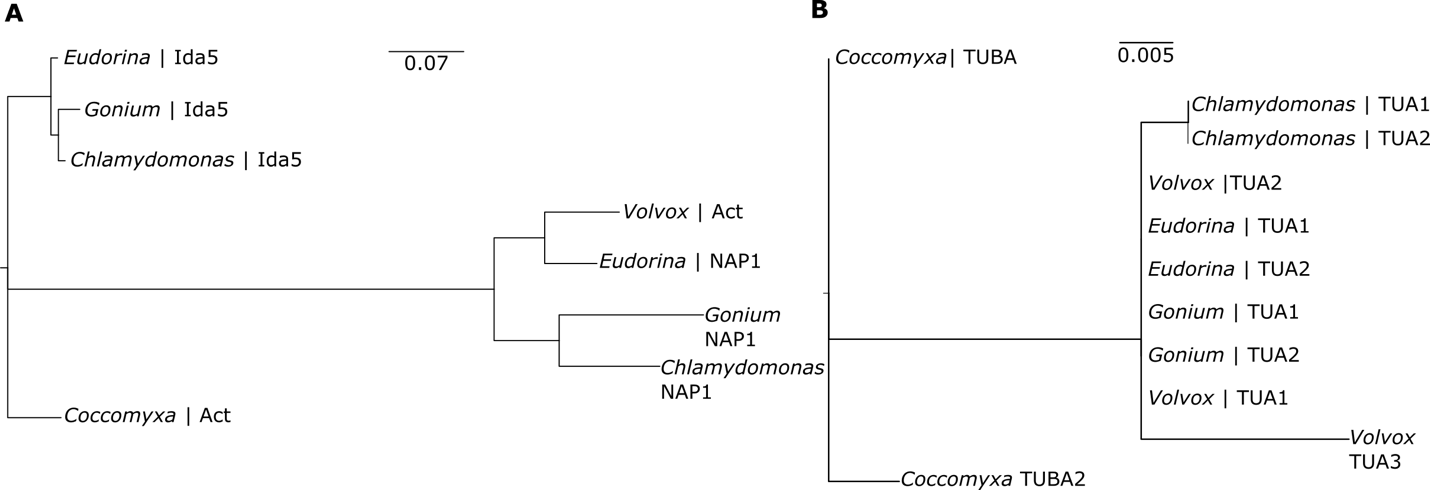


**Figure S14.** (A) β-actin and (B) α-tubulin phylogenetic trees using *Coccomyxa subellipsoidea* (Chlorophyta) as an outgroup.


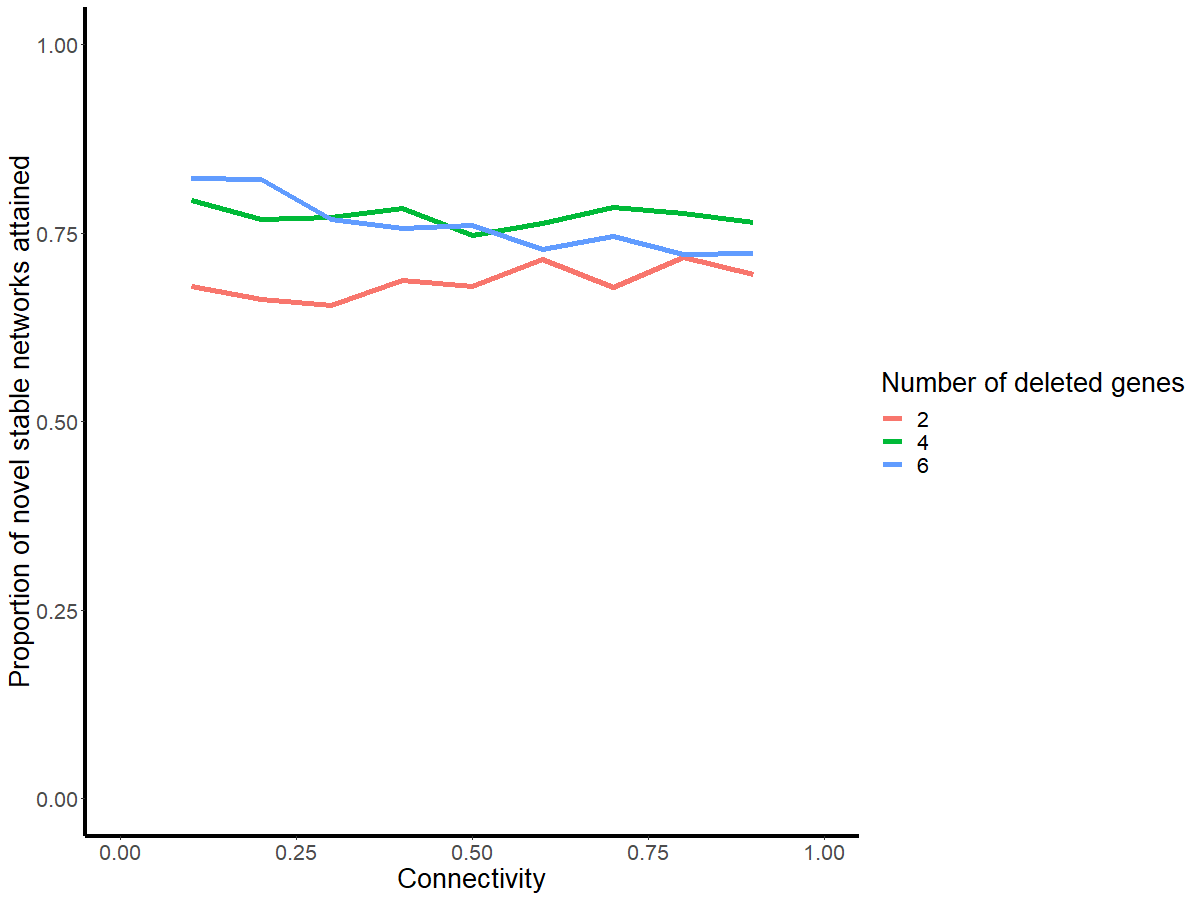


**Figure S15**. The proportion of novel stable networks following deletion of 2, 4, or 6 genes from a network of 10 genes (*N*=10) is similar, regardless of the network connectivity. For each connectivity value (ranging from low (*c*=0.1) to high (*c*=0.9)) we simulated 1000 iterations for the specified number of random deletions.


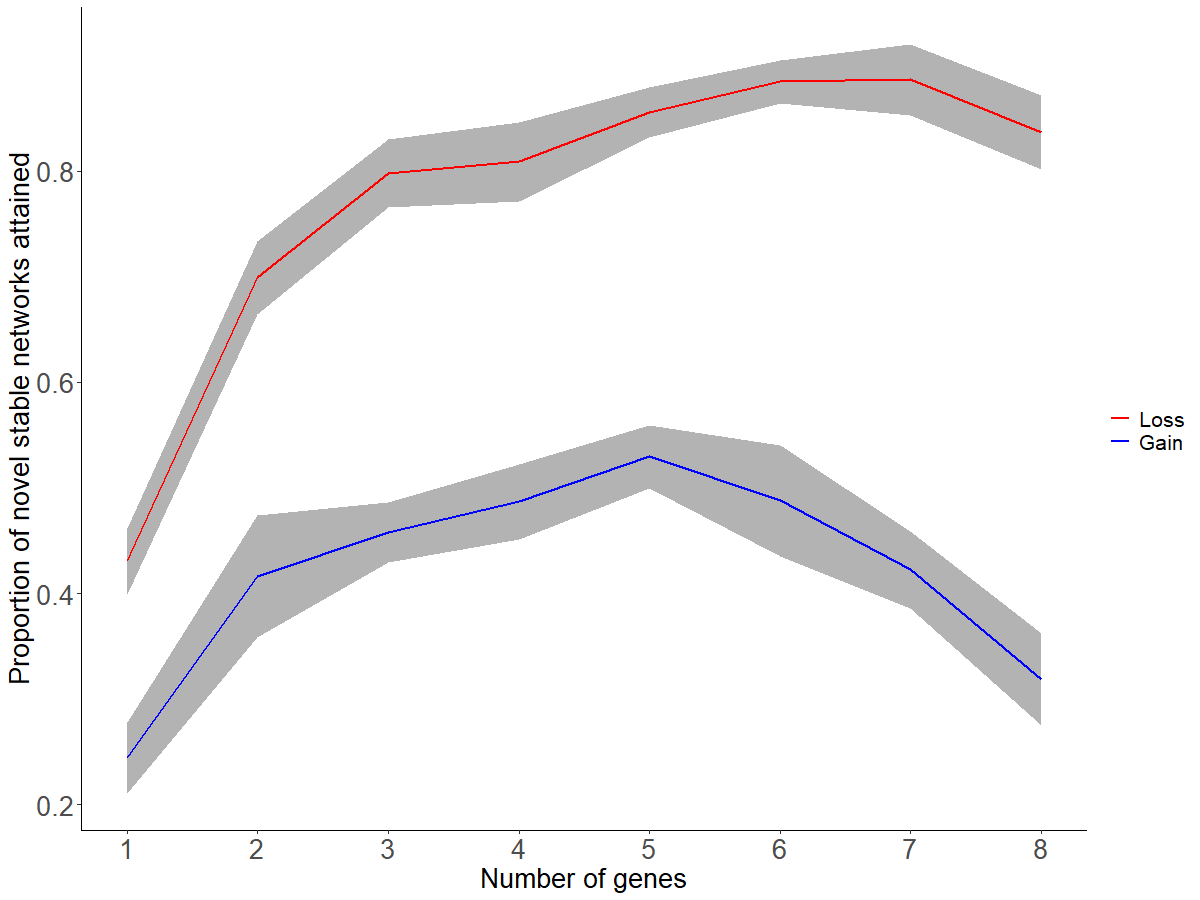


**Figure S16**. Gene loss in networks of 10 genes with random topologies that resemble real biological networks yields a higher proportion of novel stable network states than gene gain. For each simulation, we used the SeqNet R package^85^ to obtain a random adjacency matrix consistent with a reference dataset, which we then scaled by the weight from 𝑤 to obtain a new connectivity matrix. Wagner model simulates loss (red) and duplication (blue) of *k* genes. Grey shading represents variance.


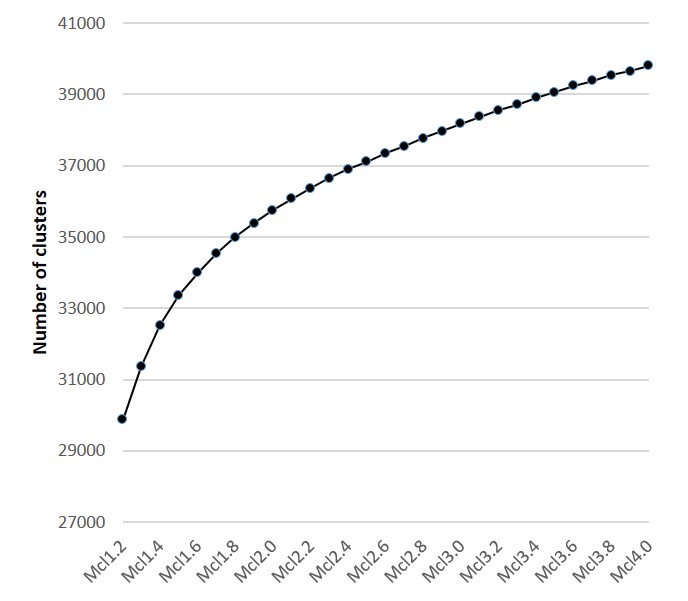


**Figure S17.** Number of orthoMCL clusters for a range of inflation values. Singletons not included.


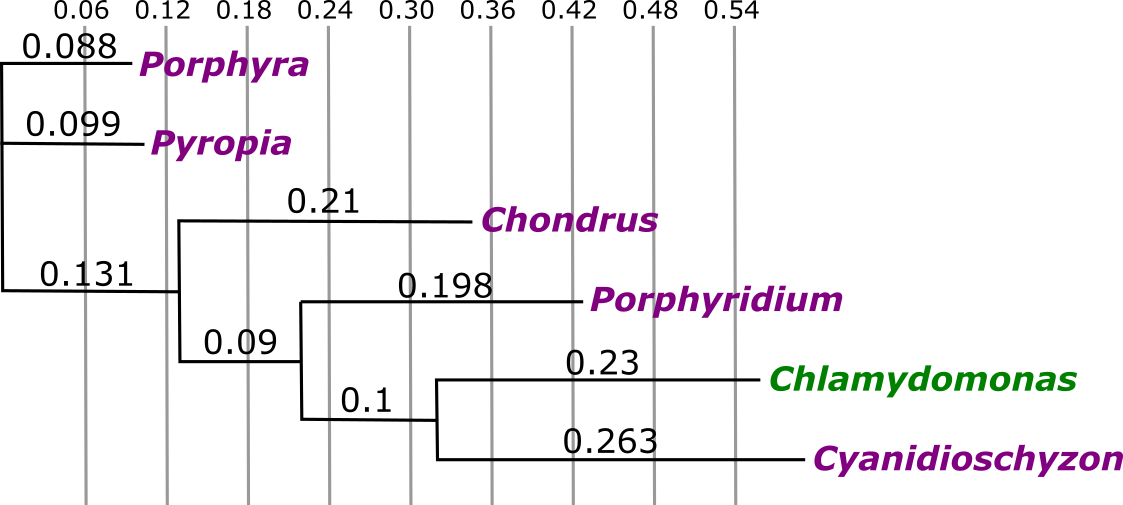


**Figure S18.** Genome-wide phylogeny of red algae (magenta) compared to a chlorophycean outgroup (green) inferred by PosiGene. Branch values indicate distance from the nearest node.

| Species | Complete (%) | Single (%) | Duplicated (%) | Fragmented (%) | Missing (%) | Total |
| --- | --- | --- | --- | --- | --- | --- |
| *Chlamydomonas* | 1503 (99) | 1411 (92.9) | 92 (6.1) | 5 (0.3) | 11 (0.7) | 1519 |
| *Tetrabaena* | 967 (63.6) | 936 (61.6) | 31 (2.0) | 277 (18.2) | 275 (18.2) | 1519 |
| *Gonium* | 1415 (93.2) | 1400 (92.2) | 15 (1) | 69 (4.5) | 35 (2.3) | 1519 |
| *Pandorina* | 1193 (78.5) | 1115 (73.4) | 78 (5.1) | 46 (3) | 280 (18.5) | 1519 |
| *Yamagishiella* | 1422 (93.6) | 992 (65.3) | 430 (28.3) | 58 (3.8) | 39 (2.6) | 1519 |
| *Eudorina* | 1340 (88.2) | 838 (55.2) | 502 (33) | 106 (7) | 73 (4.8) | 1519 |
| *Volvox* | 1464 (96.4) | 1332 (87.7) | 132 (8.7) | 16 (1.1) | 39 (2.5) | 1519 |

**Table S1.** BUSCO completeness values of seven volvocine algae annotated genomes. Completeness values describe how many BUSCOS are conserved between query genomes and the Chlorophyta database. BUSCOS are classified as Complete, Fragmented, and Missing. Complete BUSCOS can be Single or Duplicated.

|  | *Chlamydomonas* | *Gonium* | *Pandorina* | *Yamagishiella* Minus | *Yamagishiella* Plus | *Eudorina* Plus | *Eudorina* Minus | *Volvox* |
| --- | --- | --- | --- | --- | --- | --- | --- | --- |
| % G and C content | 64.1 | 64.5 | 62.8 | 62.9 | 62.8 | 63.4 | 63.2 | 56 |
| Protein coding Genes | 17741 | 17948 | 15976 | 18180 | 18416 | 20744 | 22924 | 14247 |
| Total transcripts | 19526 | 17984 | 16542 | 30755 | 31705 | 32233 | 38492 | 16075 |
| Ave Exons Per transcript | 8.62 | 6.94 | 8.67 | 7.06 | 6.93 | 6.38 | 5.73 | 7.91 |
| Average Exon length | 260.99 | 211.04 | 215.8 | 205.93 | 205.37 | 207.78 | 211.58 | 254.39 |
| Average Intron length | 279.17 | 408.89 | 457.7 | 348.37 | 348.28 | 396.73 | 382.3 | 399.5 |
| Average UTR5 length | 203.5 | 232.57 | 187.64 | 235.97 | 237.61 | 235.23 | 233.72 | 231.69 |
| Average UTR3 length | 772.74 | 711.23 | 426.37 | 702.19 | 708.88 | 725.83 | 769.03 | 1105.09 |

**Table S2.** Genomic features of six volvocine algae species**.**

| Present in | Number of orthologous groups | Number of Pfams |
| --- | --- | --- |
| C | 473 | 12 |
| G | 453 | 15 |
| P | 537 | 18 |
| Y | 317 | 11 |
| E | 411 | 17 |
| V | 226 | 23 |
| CG | 161 | 7 |
| CP | 50 | 4 |
| CY | 103 | 2 |
| CE | 91 | 1 |
| CV | 80 | 6 |
| GP | 78 | 2 |
| GY | 162 | 3 |
| GE | 120 | 5 |
| GV | 46 | 1 |
| PY | 98 | 0 |
| PE | 67 | 4 |
| PV | 33 | 0 |
| YE | 254 | 3 |
| YV | 96 | 1 |
| EV | 255 | 6 |
| CGP | 36 | 1 |
| CGY | 82 | 4 |
| CGE | 45 | 2 |
| CGV | 39 | 6 |
| CPY | 32 | 2 |
| CPE | 14 | 1 |
| CPV | 20 | 2 |
| CYE | 51 | 1 |
| CYV | 47 | 5 |
| CEV | 58 | 0 |
| GPY | 47 | 3 |
| GPE | 39 | 0 |
| GPV | 15 | 1 |
| GYE | 86 | 3 |
| GYV | 40 | 1 |
| GEV | 46 | 1 |
| PYE | 66 | 1 |
| PYV | 34 | 1 |
| PEV | 50 | 0 |
| YEV | 129 | 1 |
| CGPY | 68 | 7 |
| CGPE | 44 | 3 |
| CGPV | 26 | 12 |
| CGYE | 132 | 5 |
| CGYV | 72 | 12 |
| CGEV | 79 | 9 |
| CPYE | 84 | 1 |
| CPYV | 35 | 12 |
| CPEV | 38 | 6 |
| CYEV | 164 | 8 |
| GPYE | 88 | 3 |
| GPYV | 33 | 2 |
| GPEV | 37 | 2 |
| GYEV | 122 | 2 |
| PYEV | 124 | 3 |
| CGPYE | 354 | 31 |
| CGPYV | 145 | 68 |
| CGPEV | 120 | 43 |
| CGYEV | 1352 | 91 |
| CPYEV | 442 | 52 |
| GPYEV | 212 | 10 |
| CGPYEV | 6297 | 1184 |
| Total | 15155 | 1743 |

**Table S3.** Shared orthologous group and pfam count for six volvocine algae species. Species names are encoded as follows: C=*Chlamydomonas,* G=*Gonium,* P=*Pandorina*, Y=*Yamagishiella*, E=*Eudorina*, V=*Volvox*

| Species | Actin | mtATP-A | mtATP-B | Flagellar Dynein heavy chain | Chloroplast Ferridoxin | α-Tubulin | β-Tubulin | Flagellar Dynein Heavy Chain |
| --- | --- | --- | --- | --- | --- | --- | --- | --- |
| *Chlamydomonas* | 2 | 2 | 1 | 15 | 1 | 2 | 2 | 15 |
| *Gonium* | 2 | 3 | 1 | 15 | 1 | 2 | 2 | 15 |
| *Pandorina* | 2 | 2 | 1 | 22 | 1 | 3 | 2 | 22 |
| *Yamagishiella* | 2 | 2 | 1 | 18 | 1 | 2 | 2 | 18 |
| *Eudorina* | 2 | 2 | 1 | 16 | 1 | 2 | 2 | 16 |
| *Volvox* | 2 | 3 | 1 | 15 | 2 | 2 | 2 | 15 |

**Table S4.** Conserved gene counts for six volvocine species.

| Species | H1 |
| --- | --- |
| *Chlamydomonas* | 3 |
| *Gonium* | 3 |
| *Pandorina* | 2 |
| *Yamagishiella* | 3 |
| *Eudorina* | 2 |
| *Volvox* | 4 |

**Table S5.** Histone H1 gene counts for volvocine algae.

| Histone | Species | Copy number | Tail var. number |
| --- | --- | --- | --- |
| H2A | *Chlamydomonas* | 31 | 5 |
|  | *Gonium* | 34 | 5 |
|  | *Pandorina* | 30 | 1 |
|  | *Yamagishiella* | 19 | 3 |
|  | *Eudorina* | 19 | 4 |
|  | *Volvox* | 15 | 3 |
| H2B | *Chlamydomonas* | 29 | 9 |
|  | *Gonium* | 35 | 12 |
|  | *Pandorina* | 32 | 9 |
|  | *Yamagishiella* | 20 | 13 |
|  | *Eudorina* | 18 | 14 |
|  | *Volvox* | 15 | 12 |
| H3 | *Chlamydomonas* | 35 | 4 |
|  | *Gonium* | 30 | 24 |
|  | *Pandorina* | 30 | 1 |
|  | *Yamagishiella* | 21 | 6 |
|  | *Eudorina* | 23 | 4 |
|  | *Volvox* | 13 | 3 |
| H4 | *Chlamydomonas* | 32 | 2 |
|  | *Gonium* | 34 | 3 |
|  | *Pandorina* | 30 | 1 |
|  | *Yamagishiella* | 27 | 3 |
|  | *Eudorina* | 21 | 2 |
|  | *Volvox* | 14 | 2 |

**Table S6.** Histone family copy number and tail variant number of six volvocine algae.

| Species | H1 | H2A | H2B | H3 | H4 |
| --- | --- | --- | --- | --- | --- |
| *Porphyra* | 1 | 6 | 4 | 74 | 2 |
| *Pyropia* | 1 | 3 | 1 | 22 | 1 |
| *Chondrus* | 1 | 3 | 2 | 21 | 3 |
| *Porphyridium* | 1 | 3 | 2 | 3 | 2 |
| *Cyanidioschyzon* | 1 | 3 | 2 | 2 | 2 |

**Table S7.** Histone gene counts for Rhodophytes.

| Absence in | Total | Decay | Deletion |
| --- | --- | --- | --- |
| gpyev | 1017 | 880 | 137 |
| gpye | 0 | 0 | 0 |
| gpyv | 8 | 6 | 2 |
| gpev | 2 | 1 | 1 |
| gyev | 5 | 5 | 0 |
| pyev | 30 | 26 | 4 |
| gpy | 0 | 0 | 0 |
| gpe | 0 | 0 | 0 |
| gpv | 2 | 2 | 0 |
| gye | 1 | 1 | 0 |
| gyv | 0 | 0 | 0 |
| gev | 1 | 1 | 0 |
| pye | 1 | 1 | 0 |
| pyv | 8 | 5 | 3 |
| pev | 6 | 4 | 2 |
| yev | 8 | 6 | 2 |
| gp | 1 | 1 | 0 |
| gy | 0 | 0 | 0 |
| py | 0 | 0 | 0 |
| pe | 2 | 1 | 1 |
| pv | 6 | 6 | 0 |
| ge | 0 | 0 | 0 |
| ye | 0 | 0 | 0 |
| gv | 2 | 1 | 1 |
| ev | 11 | 11 | 0 |
| yv | 5 | 3 | 2 |
| g | 3 | 3 | 0 |
| p | 4 | 3 | 1 |
| y | 3 | 2 | 1 |
| e | 1 | 1 | 0 |
| v | 14 | 5 | 9 |

**Table S8.** Distribution and type of gene losses in multicellular volvocine algae. Species names are encoded as follows: G=*Gonium,* P=*Pandorina*, Y=*Yamagishiella*, E=*Eudorina*, V=*Volvox.* All values have a P-value<0.01 and reject uniform distribution of decay and deletion events.

| Species | MMP genes | Pherophorin genes |
| --- | --- | --- |
| *Chlamydomonas* | 34 | 41 |
| *Gonium* | 22 | 21 |
| *Pandorina* | 42 | 38 |
| *Yamagishiella* | 38 | 32 |
| *Eudorina* | 63 | 69 |
| *Volvox* | 58 | 68 |

**Table S9.** Matrix metalloprotease (MMP) and pherophorin gene counts for six volvocine species.
