# Supplementary material for: Gene loss during a transition to multicellularity
Source: Sci Rep. 2023 Mar 31;13:5268. doi: 10.1038/s41598-023-29742-2 (PMC10066295; doi:10.1038/s41598-023-29742-2)
Supplement: Supplementary file 2 — Supplementary Information 2. [file 41598_2023_29742_MOESM2_ESM.docx]

Supplementary Files can be accessed at <https://jimenezbere@bitbucket.org/jimenezbere/supplementary_files.git>

Custom scripts can be accessed at

<https://jimenezbere@bitbucket.org/jimenezbere/orthotrends.git> <https://jimenezbere@bitbucket.org/jimenezbere/group2gene.git> <https://jimenezbere@bitbucket.org/jimenezbere/synteny_suite.git> https://jimenezbere@bitbucket.org/jimenezbere/wagner_extended_model.git
